# Higher expression of denervation‐responsive genes is negatively associated with muscle volume and performance traits in the study of muscle, mobility, and aging (SOMMA)

**DOI:** 10.1111/acel.14115

**Published:** 2024-06-03

**Authors:** Cole J. Lukasiewicz, Gregory J. Tranah, Daniel S. Evans, Paul M. Coen, Haley N. Barnes, Zhiguang Huo, Karyn A. Esser, Xiping Zhang, Christopher Wolff, Kevin Wu, Nancy E. Lane, Steven B. Kritchevsky, Anne B. Newman, Steven R. Cummings, Peggy M. Cawthon, Russell T. Hepple

**Affiliations:** ^1^ Department of Physical Therapy University of Florida Gainesville Florida USA; ^2^ California Pacific Medical Center Research Institute San Francisco California USA; ^3^ Department of Epidemiology and Biostatistics University of California San Francisco California USA; ^4^ Translational Research Institute, Advent Health Orlando Florida USA; ^5^ Department of Physiology and Aging University of Florida College of Medicine Gainesville Florida USA; ^6^ Department of Medicine, Division of Rheumatology University of California Davis Health Sacramento California USA; ^7^ Department of Internal Medicine Wake Forest University School of Medicine Winston‐Salem North Carolina USA; ^8^ School of Public Health University of Pittsburgh Pittsburgh Pennsylvania USA

**Keywords:** gene expression profiling, muscle, skeletal, neuromuscular junction, denervation

## Abstract

With aging skeletal muscle fibers undergo repeating cycles of denervation and reinnervation. In approximately the 8th decade of life reinnervation no longer keeps pace, resulting in the accumulation of persistently denervated muscle fibers that in turn cause an acceleration of muscle dysfunction. The significance of denervation in important clinical outcomes with aging is poorly studied. The Study of Muscle, Mobility, and Aging (SOMMA) is a large cohort study with the primary objective to assess how aging muscle biology impacts clinically important traits. Using transcriptomics data from vastus lateralis muscle biopsies in 575 participants we have selected 49 denervation‐responsive genes to provide insights to the burden of denervation in SOMMA, to test the hypothesis that greater expression of denervation‐responsive genes negatively associates with SOMMA participant traits that included time to walk 400 meters, fitness (VO_2peak_), maximal mitochondrial respiration, muscle mass and volume, and leg muscle strength and power. Consistent with our hypothesis, increased transcript levels of: a calciumdependent intercellular adhesion glycoprotein (CDH15), acetylcholine receptor subunits (CHRNA1, CHRND, CHRNE), a glycoprotein promoting reinnervation (NCAM1), a transcription factor regulating aspects of muscle organization (RUNX1), and a sodium channel (SCN5A) were each negatively associated with at least 3 of these traits. VO_2peak_ and maximal respiration had the strongest negative associations with 15 and 19 denervation‐responsive genes, respectively. In conclusion, the abundance of denervationresponsive gene transcripts is a significant determinant of muscle and mobility outcomes in aging humans, supporting the imperative to identify new treatment strategies to restore innervation in advanced age.

## Graphical abstract

We used RNAseq to assess how the abundance of 49 denervation‐responsive transcripts relates to physical function traits in the Study of Muscle, Mobility, and Aging (SOMMA). Generally, we found that elevated expression of denervation‐responsive transcripts had negative associations with walking speed, VO2peak, mitochondrial respiratory capacity, leg strength, and leg power, suggesting that denervation is an important contributor to declining function in aging humans.

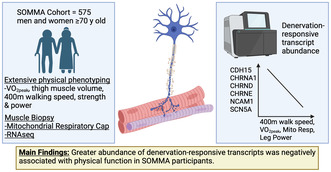

## 1 INTRODUCTION

During the aging process skeletal muscle fibers undergo repeating cycles of denervation that are usually followed by successful reinnervation (Hepple & Rice, 2016). This process profoundly remodels the spatial distribution of muscle fibers within the motor unit because a denervated muscle fiber is likely to be reinnervated by an axon sprout from the immediately adjacent muscle fibers. This results in the muscle fibers belonging to a given motor unit being more likely to be adjacent to one another with aging (Larsson, 1995). Beyond this, the most significant impact of these recurring cycles of denervation‐reinnervation is that eventually the rate of reinnervation cannot keep pace with denervation, resulting in the accumulation of muscle fibers that lack connection to a motor neuron (Anagnostou & Hepple, 2020). These persistently denervated muscle fibers progressively atrophy over time and can no longer contribute to force generation. For these reasons the accumulation of these persistently denervated fibers is an important contributor to both the reduction in muscle mass and contractile dysfunction with aging.

Rodent model studies find that alterations in neuromuscular junction morphology can be seen in some muscles relatively early in the lifespan (adulthood) (Burke et al., 2021; Valdez et al., 2010), and this occurs well in advance of the accumulation of persistently denervated muscle fibers and the presentation of muscle atrophy with aging (Deschenes et al., 2010). Indeed, the accumulation of persistently denervated muscle fibers is a relatively late occurrence that can progress quite rapidly in advanced age (≥75 years old in humans), and impacts various skeletal muscle phenotypes (Rowan et al., 2012) including mitochondrial function (Sonjak, Jacob, Spendiff, et al., 2019; Spendiff et al., 2016). Studies in rodent models of experimental denervation, such as sciatic nerve transection, have identified genes that respond (either increasing or decreasing depending upon the gene) to denervation and interestingly, many of these genes are also seen to change in aging muscle. For example, genes that regulate the stability of the acetylcholine receptor (AChR) cluster at the neuromuscular junction (e.g., muscle specific kinase [MuSK], AChR subunits, Agrin, rapsyn) are often seen to increase dramatically with surgical denervation (Ebert et al., 2012; Macpherson et al., 2015; Mugahid et al., 2019; Soares et al., 2014), and many of these genes are also seen to increase in rodent (Aare et al., 2016; Barns et al., 2014; Ibebunjo et al., 2013) and human skeletal muscle (Kang et al., 2021; Spendiff et al., 2016) with aging. Hence, analysis of the expression levels of denervation‐responsive genes can provide an indicator of the denervation burden in aging muscle. A significant question, however, concerns the importance of denervation in the decline in mobility with aging.

Previous studies examining how alterations in expression of denervation/neuromuscular junction genes are impacted with aging in humans have typically been small and have not had the power to establish how these alterations might relate to clinically important changes in physical function with aging. In this respect, the Study of Muscle, Mobility and Aging (SOMMA) is a large cohort study with the primary objective to assess how features of aging muscle biology relate to fitness, strength, muscle mass and other clinically relevant traits (Cummings et al., 2023). Part of this study has involved acquiring muscle biopsies from the vastus lateralis muscle. Amongst the battery of measures performed with these biopsy specimens is whole transcriptome analysis using RNAseq. In this manuscript, using transcriptomics data from 575 participants we have selected 49 denervation‐responsive genes to provide insights to the burden of denervation and test the hypothesis that expression levels of denervation‐responsive genes are associated with walking speed, muscle mass, muscle strength/power, and fitness (VO_2peak_). The 49 denervation‐responsive genes exhibit increased expression levels in surgical denervation studies (Ebert et al., 2012; Macpherson et al., 2015; Mugahid et al., 2019; Soares et al., 2014) and have previously been shown to be enriched amongst the differentially expressed genes seen in aging rat skeletal muscle (Ibebunjo et al., 2013). These include genes involved in maintenance of the AChR cluster (AGRN, LRP4, MUSK, RPSN), genes encoding the 5 AChR subunits (CHRNA, CHRNB, CHRND, CHRNE, CHRNG), genes promoting reinnervation (NCAM1, NRG1), genes encoding different isoforms of sodium channels (SCN4A, SCN5A), genes involved in the denervation atrophy pathway (MYOG, GADD45A), and other genes that are enriched at the muscle endplate and which increase in response to denervation.

## 2 RESULTS

### 2.1 Participant characteristics

Of the 879 participants who completed baseline measures, 591 had RNA sequencing completed and of these 575 had high quality sequencing and a complete set of covariate data for planned analyses. The characteristics of these 575 participants are provided in Table 1.

**TABLE 1:** Baseline characteristics of included SOMMA participants stratified by tertiles of VO_2_peak.

Variable	Total *N*	Total summary	Tertile 1	Tertile 2	Tertile 3	*p*‐ Value	*p*‐ Trend
			653 <=T1 < 1327	1327 <=T2 < 1705	1705 <=T3 < 3195		
		(*N* = 575)	(*N* = 182)	(*N* = 185)	(*N* = 184)		
Clinic Site: Pittsburgh	575	263 (45.7)	81 (44.5)	86 (46.5)	89 (48.4)	0.760	0.459
Age, years	575	75.9 ± 4.5	77.3 ± 4.8	75.4 ± 4.4	74.9 ± 3.8	<0.001	<0.001
Sex: Female	575	319 (55.5)	165 (90.7)	121 (65.4)	17 (9.2)	<0.001	<0.001
Race: Non‐Hispanic White	575	503 (87.5)	158 (86.8)	163 (88.1)	165 (89.7)	0.697	0.397
Height, m	575	1.7 ± 0.1	1.6 ± 0.1	1.7 ± 0.1	1.8 ± 0.1	<0.001	<0.001
Weight, kg	575	76.2 ± 15.5	66.3 ± 11.0	75.3 ± 13.3	86.8 ± 14.4	<0.001	<0.001
CHAMPS: Hours/week in all exercise‐related activities	575	15.7 ± 11.4	13.7 ± 10.1	15.5 ± 9.7	18.5 ± 13.8	0.001	<0.001
Multimorbidity count	575					0.394	0.200
0 Chronic conditions		254 (44.2)	79 (43.4)	76 (41.1)	90 (48.9)		
1 Chronic condition		220 (38.3)	71 (39.0)	72 (38.9)	70 (38.0)		
2+ Chronic conditions		101 (17.6)	32 (17.6)	37 (20.0)	24 (13.0)		
Maximal respiration (pmol/(s*mg))	519	61.5 ± 18.1	53.4 ± 13.5	62.5 ± 15.8	69.1 ± 20.9	<0.001	<0.001
Leg Strength: 1 repetition maximum	559	177.9 ± 62.1	135.4 ± 37.4	171.8 ± 43.7	229.9 ± 61.0	<0.001	<0.001
400 m walk speed (m/s)	575	1.9 ± 0.2	1.0 ± 0.2	1.1 ± 0.2	1.1 ± 0.2	<0.001	<0.001
Total thigh fat free muscle vol, L	557	9.1 ± 2.3	7.2 ± 1.2	8.8 ± 1.5	11.6 ± 1.6	<0.001	<0.001
D_3_CR muscle mass (kg)	551	22.6 ± 6.6	18.0 ± 3.6	21.6 ± 5.2	28.2 ± 6.1	<0.001	<0.001

*Note*: Data shown as *n* (%), mean ± Standard Deviation. *p*‐values are presented for variables across tertiles of VO_2_ peak. *p*‐values for continuous variables from ANOVA for normally distributed data, a KruskalWallis test for skewed data. P for linear trend across categories was calculated with linear regression models for those normally distributed variables, a Jonckheere‐Terpstra test for skewed data. P‐values for categorical data from a chi‐square test for homogeneity. P for trend was calculated with the Jonckheere‐Terpstra test.

### 2.2 RNA (human Ensembl genes (ENSG)) detection

The mean, median, and SD of the PCR duplicate percent per sample was 59%, 56%, and 9%, respectively (Table S1). After PCR duplicates were removed, the number of aligned reads per sample was high (mean = 69,117,209, median = 71,313,059, SD = 14,444,848, range = 12,853,785‐102,724,183).

### 2.3 Associations with age and other SOMMA participant traits

**FIGURE 1:**
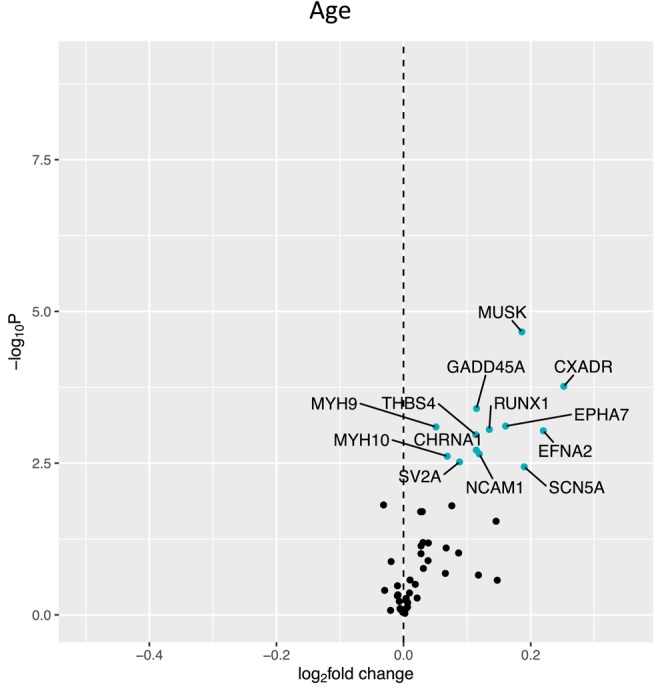
Associations with Age. Volcano plot capturing all statistically significant (*p* < 0.05 FDR adjusted) genes identified by our models: Each dot represents a gene; blue dots reached an adjusted *p* < 0.05 threshold. Base model: gene expression~age + sex + clinic site + race/ethnicity + body size + CHAMPS + multimorbidity count + sequencing batch.

**FIGURE 2:**
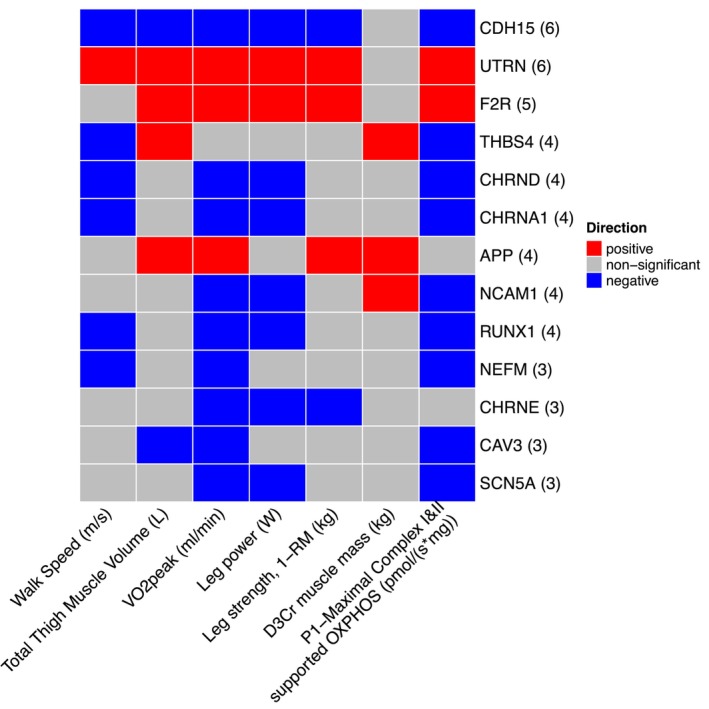
Significant associations with mitochondrial function, physical performance, and muscle mass measures. Heat map capturing all statistically significant (*p* < 0.05 FDR adjusted) genes demonstrating associations with at least 3 traits identified by our models: each color represents positive (red) or negative (blue) associations.

**FIGURE 3:**
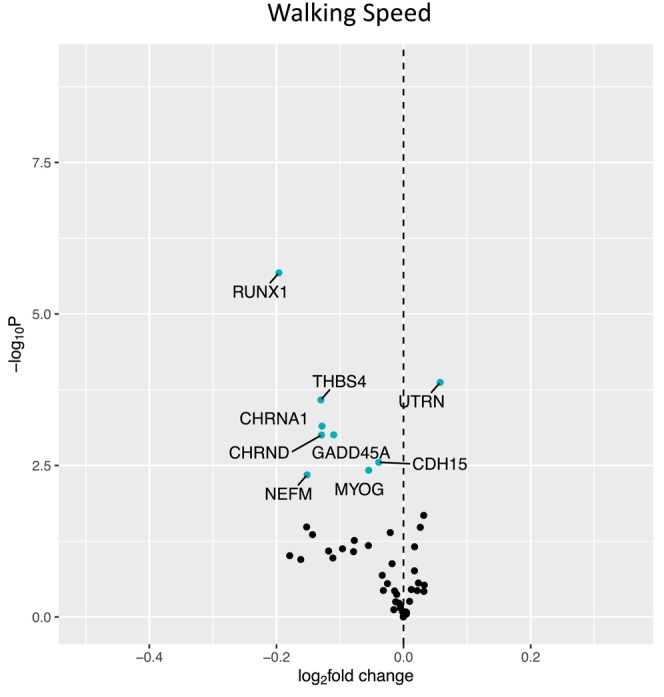
Associations with 400 m Walk Speed. Volcano plot capturing all statistically significant (*p* < 0.05 FDR adjusted) genes identified by our models: Each dot represents a gene; blue dots reached an adjusted *p* < 0.05 threshold. Base model: gene expression~walk speed + age + sex + clinic site + race/ethnicity + body size + CHAMPS + multimorbidity count + sequencing batch.

**FIGURE 4:**
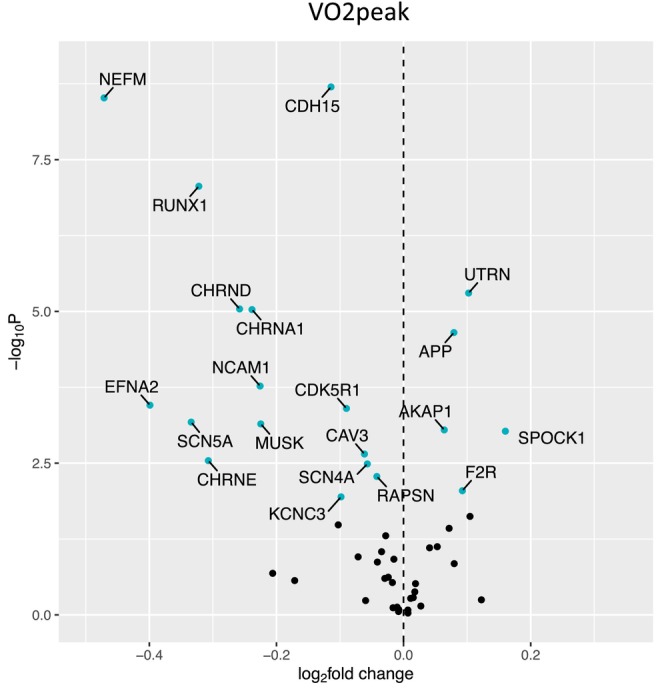
Associations with VO_2_ Peak. Volcano plot capturing all statistically significant (*p* < 0.05 FDR adjusted) genes identified by our models: Each dot represents a gene; blue dots reached an adjusted *p* < 0.05 threshold. Base model: gene expression~VO_2_ peak + age + sex + clinic site + race/ethnicity + body size + CHAMPS + multimorbidity count + sequencing batch.

**FIGURE 5:**
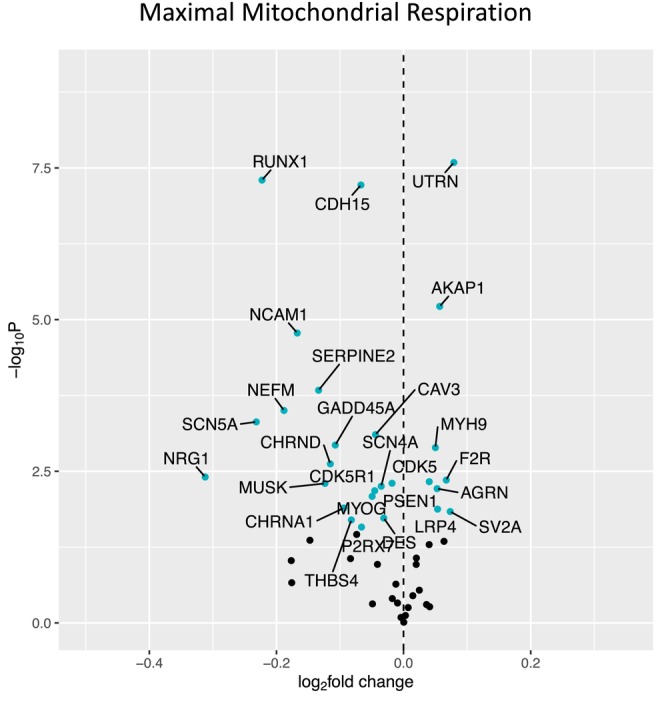
Associations with Maximal Mitochondrial Respiration. Volcano plot capturing all statistically significant (*p* < 0.05 FDR adjusted) genes identified by our models: Each dot represents a gene; blue dots reached an adjusted P < 0.05 threshold. Base model: gene expression~maximal respiration + age + sex + clinic site + race/ethnicity + body size + CHAMPS + multimorbidity count + sequencing batch.

**FIGURE 6:**
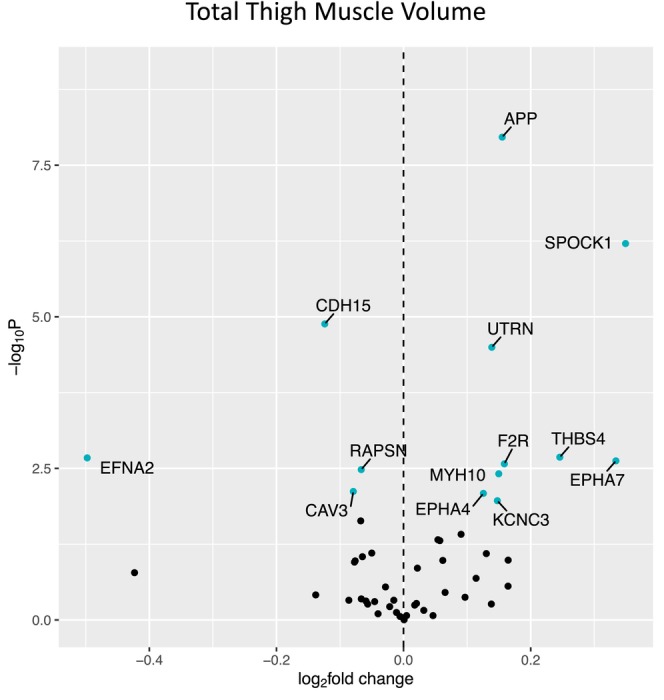
Associations with Total Thigh Muscle Volume. Volcano plot capturing all statistically significant (*p* < 0.05 FDR adjusted) genes identified by our models: Each dot represents a gene; blue dots reached an adjusted *p* < 0.05 threshold. Base model: gene expression~thigh muscle volume + age + sex + clinic site + race/ethnicity + body size + CHAMPS + multimorbidity count + sequencing batch.

**FIGURE 7:**
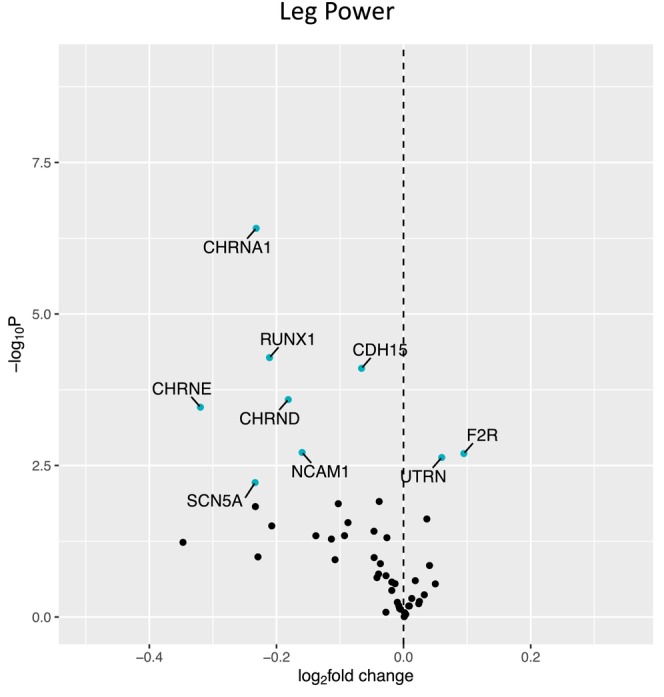
Associations with Leg Power. Volcano plot capturing all statistically significant (*p* < 0.05 FDR adjusted) genes identified by our models: Each dot represents a gene; blue dots reached an adjusted *p* < 0.05 threshold. Base model: gene expression~leg power + age + sex + clinic site + race/ethnicity + body size + CHAMPS + multimorbidity count + sequencing batch.

**FIGURE 8:**
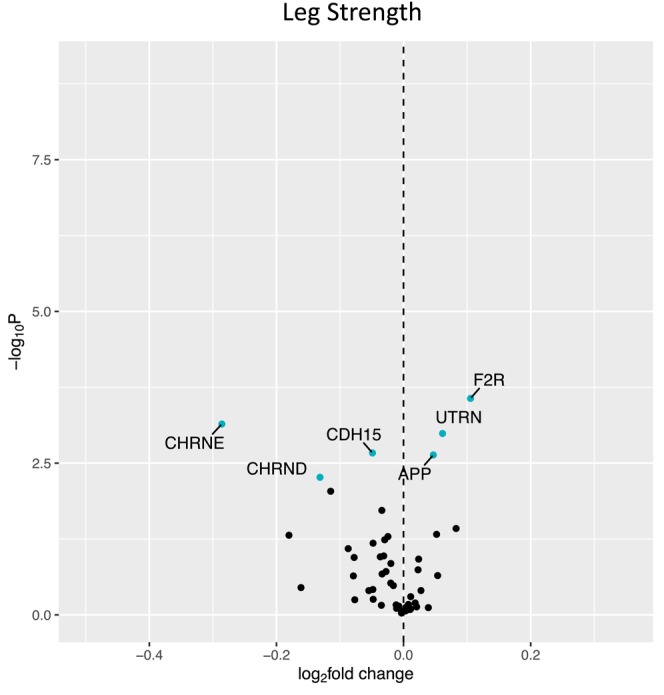
Associations with Leg Strength. Volcano plot capturing all statistically significant (*p* < 0.05 FDR adjusted) genes identified by our models: Each dot represents a gene; blue dots reached an adjusted *p* < 0.05 threshold. Base model: gene expression~leg strength + age + sex + clinic site + race/ethnicity + body size + CHAMPS + multimorbidity count + sequencing batch.

Figure 1 shows a volcano plot to illustrate the positive association between older age (at 5 years intervals) and greater expression of 13 denervation‐responsive genes, where genes that satisfied an adjusted *p* < 0.05 are indicated in blue. We then characterized the association of SOMMA traits with the denervation‐responsive genes reporting log base 2‐fold changes reflecting the change in gene expression per one SD unit change in each trait. A summary of those genes that associated with at least three SOMMA traits is shown in a heatmap in Figure 2. Most of these associations were in the negative direction (an increase in transcript abundance associated with a lower value for a given trait); however, there were several transcripts for which we observed positive relationships between higher gene expression and a higher value for a given trait. This was most notable for UTRN which exhibited a positive association with six different traits. Notably, 3 of the genes with at least three significant negative associations encoded for structural components of the AChR cluster at the neuromuscular junction (CHRNA1, CHRND, CHRNE), one for a glycoprotein which helps attract neighboring axons to reinnervate denervated muscle fibers (NCAM1), and another encoded a sodium channel isoform found in muscle developmentally and which typically only appears in adult muscle following denervation (SCN5A). Turning to the specific trait associations, Figure 3 shows a Volcano plot of the genes that associated with walking speed. Of the nine genes that reached an adjusted *p* < 0.05, eight exhibited a negative association (RUNX1, THBS4, CHRNA1, CHRND, GADD45A, CDH15, NEFM, and MYOG), whereas UTRN had a positive association with walking speed. VO_2peak_ (Figure 4) and maximal mitochondrial respiration (Figure 5) each demonstrated associations with the most denervation‐responsive genes of all the traits examined and shared a common set of 11 genes for which there were negative associations (NEFM, CDH15, RUNX1, CHRNA1, CHRND, NCAM1, MUSK, CDK5R1, CAV3, SCN4A, and SCN5A). Total thigh muscle volume demonstrated negative associations with 4 denervation‐responsive genes, but positive associations with 9 denervation‐responsive genes (Figure 6). Leg power demonstrated negative associations with 7 denervation‐responsive genes (Figure 7), whereas leg strength demonstrated negative associations with 3 denervation‐responsive genes (Figure 8). We found no significant associations between any of the denervation‐responsive genes and D3Cr total body muscle mass. A summary of the location and/or function of each of the genes that showed a significant negative or positive association with any of the participant traits is found in Tables 2 and 3.

**TABLE 2:** Genes with which SOMMA traits had a negative association.

Gene name	Localization	Citations
CDH15	A calcium‐dependent intercellular adhesion glycoprotein found on muscle fiber side of adult neuromuscular junction	1. https://pubmed.ncbi.nlm.nih.gov/8921257/ 2. https://pubmed.ncbi.nlm.nih.gov/9638336/
THBS4	Matricellular protein found at adult neuromuscular junction	1. https://pubmed.ncbi.nlm.nih.gov/7490284/
CHRND	Delta subunit of acetylcholine receptor found on muscle fiber side of adult neuromuscular junction	1. https://pubmed.ncbi.nlm.nih.gov/1646821/ 2. https://pubmed.ncbi.nlm.nih.gov/8300573/
CHRNA1	Alpha subunit of acetylcholine receptor found on muscle fiber side of adult neuromuscular junction	1. https://pubmed.ncbi.nlm.nih.gov/1646821/ 2. https://pubmed.ncbi.nlm.nih.gov/8300573/
CHRNE	Epsilon subunit of acetylcholine receptor found on muscle fiber side of adult neuromuscular junction	1. https://pubmed.ncbi.nlm.nih.gov/1646821/ 2. https://pubmed.ncbi.nlm.nih.gov/8300573/
NCAM1	Glycoprotein found on both muscle fiber and axonal sides of neuromuscular junction	1. https://pubmed.ncbi.nlm.nih.gov/29175953/
RUNX1	Transcription factor found in myonuclei	1. https://pubmed.ncbi.nlm.nih.gov/16024660/
NEFM	A neurofilament protein typically found in axons. Gene has very low baseline expression in skeletal muscle and protein is not detected	1. https://www.proteinatlas.org/ENSG00000104722NEFM/tissue
CAV3	The primary protein structural component of caveolae in skeletal muscle	1. https://www.nature.com/articles/ejhg2009103
SCN5A	A sodium channel that is constitutively expressed in adult heart, and although expressed in neonatal skeletal muscle, is typically not expressed in adult skeletal muscle except when denervated	1. https://pubmed.ncbi.nlm.nih.gov/2155010/ 2. https://pubmed.ncbi.nlm.nih.gov/1654949/
SCN4A	A sodium channel that is expressed in adult skeletal muscle	1. https://pubmed.ncbi.nlm.nih.gov/2155010/ 2. https://pubmed.ncbi.nlm.nih.gov/1654949/
MYOG	A transcription factor that regulates aspects of muscle regeneration, as well as regulating expression of ubiquitin ligases (e.g., atrogin1/MAFbx) with denervation	1. https://pubmed.ncbi.nlm.nih.gov/20887891/
GADD45A	Transcription factor that is upregulated in muscle atrophy conditions that include denervation	1. https://pubmed.ncbi.nlm.nih.gov/22692,209/ 2. https://pubmed.ncbi.nlm.nih.gov/23941879/
EFNA2	Membrane bound protein whose expression increases in injured Schwann cells	1. https://pubmed.ncbi.nlm.nih.gov/32349983/
CDK5R1	Receptor for the kinase cdk5 expressed in muscle satellite cells	1. https://pubmed.ncbi.nlm.nih.gov/28733456/
MUSK	Muscle Specific Kinase found in skeletal muscle membrane and responsible for recruiting proteins to subsynaptic region to anchor acetylcholine receptors to Actin cytoskeleton	1. https://pubmed.ncbi.nlm.nih.gov/21255125/ 2. https://pubmed.ncbi.nlm.nih.gov/209,74278/
RAPSN	A protein that helps anchor acetylcholine receptors to Actin cytoskeleton at neuromuscular junction.	1. https://pubmed.ncbi.nlm.nih.gov/32344,108/
KCNC3	A potassium channel found in various tissues that include skeletal muscle	1. https://www.proteinatlas.org/ENSG00000131398KCNC3/tissue#rnaexpression
SERPINE2	Serine protease inhibitor. Gene is expressed in skeletal muscle but protein not detected	1. https://www.proteinatlas.org/ENSG00000135919‐SERPINE2/tissue
NRG1	Growth factor implicated in regulating acetylcholine receptor number at neuromuscular junction	1. https://www.ncbi.nlm.nih.gov/pmc/articles/PMC5864756/
P2RX7	Purinergic receptor involved in formation and function of the neuromuscular junction.	1. https://www.mdpi.com/14220067/24/11/9434
DES	An intermediate filament protein linking contractile proteins to the sarcolemma and organelles	1. https://pubmed.ncbi.nlm.nih.gov/33825,342/

**TABLE 3:** Genes with which SOMMA traits had a positive association.

Gene name	Localization of protein	Citations
UTRN	A protein structurally and functionally similar to dystrophin expressed primarily in neuromuscular and myotendinous junctions	1.https://pubmed.ncbi.nlm.nih.gov/24879867/ 2.https://www.ncbi.nlm.nih.gov/gene/7402 3.https://pubmed.ncbi.nlm.nih.gov/10446253/
F2R	G‐protein‐coupled receptor expressed in osteoclasts and skeletal muscle	1. https://pubmed.ncbi.nlm.nih.gov/32226307/ 2. https://www.proteinatlas.org/ENSG00000181104F2R/tissue
APP	A transmembrane protein expressed at both the pre and post‐synaptic sides of the NMJ	1. https://pubmed.ncbi.nlm.nih.gov/37175515/ 2. https://pubmed.ncbi.nlm.nih.gov/15689559/
AKAP1	A scaffolding protein found in the mitochondria	1. https://pubmed.ncbi.nlm.nih.gov/26437602/ 2. https://pubmed.ncbi.nlm.nih.gov/26610411/
SPOCK1	A proteoglycan predominately expressed in axons and Schwann cells in adults. Gene expressed in muscle but protein not detected	1. https://pubmed.ncbi.nlm.nih.gov/10842087/ 2. https://www.proteinatlas.org/ENSG00000152377SPOCK1/tissue
MYH9	A nonmuscle myosin found in cytoplasm assisting in cytoskeleton maintenance	1.https://pubmed.ncbi.nlm.nih.gov/29679756/
AGRN	Proteoglycan found in the basal lamina of the NMJ in both skeletal muscle and alpha motor neurons	1. https://pubmed.ncbi.nlm.nih.gov/28846617/ 2. https://pubmed.ncbi.nlm.nih.gov/12798796/
SV2A	Synaptic vesicle protein found in the nerve terminals of type I and smaller IIa fibers. Although gene is expressed in skeletal muscle, protein is not detected	1. https://pubmed.ncbi.nlm.nih.gov/20843861/ 2. https://www.proteinatlas.org/ENSG00000159164‐SV2A/tissue.
LRP4	Agrin receptor found in the skeletal muscle membrane at NMJ	1. https://www.ncbi.nlm.nih.gov/pmc/articles/PMC7759742/pdf/fnagi‐12‐597,811.pdf
CDK5	A kinase concentrated at the neuromuscular synapse.	1. https://pubmed.ncbi.nlm.nih.gov/11276227/
THBS4	An adhesive glycoprotein that is a member of thrombospondin protein family. Stabilizes muscle membranes	1. https://pubmed.ncbi.nlm.nih.gov/37582915/ 2. https://pubmed.ncbi.nlm.nih.gov/24589453/ 3. https://pubmed.ncbi.nlm.nih.gov/27669143/
PSEN1	A protein needed for normal neurogenesis and may have anti‐apoptotic activity	1. https://www.ncbi.nlm.nih.gov/pmc/articles/PMC9504248/pdf/ijms‐23‐10,970.pdf
		2. https://www.ncbi.nlm.nih.gov/pmc/articles/PMC10179041/
		3. https://www.proteinatlas.org/ENSG00000080815‐PSEN1/tissue
EPHA7	A juxtacrine signaling receptor concentrated in the post‐synaptic membrane of muscle fibers and promotes muscle differentiation	1. https://pubmed.ncbi.nlm.nih.gov/11414792/ 2. https://pubmed.ncbi.nlm.nih.gov/32314958/
KCNC3	Potassium channel expressed in various CNS neurons, especially purkinje cells, with lower expression in skeletal muscle	1. https://pubmed.ncbi.nlm.nih.gov/18448641/ 2. https://pubmed.ncbi.nlm.nih.gov/34820911/ 3. https://www.proteinatlas.org/ENSG00000131398‐KCNC3/tissue
EPHA4	An ephrin receptor concentrated in the postsynaptic membrane of muscle fibers that is implicated in formation and stability of neuromuscular junction	1. https://pubmed.ncbi.nlm.nih.gov/11414792/ 2. https://pubmed.ncbi.nlm.nih.gov/14729671/
MYH10	A nonmuscle myosin isoform. Gene is expressed in skeletal muscle but protein is not detected	1. https://pubmed.ncbi.nlm.nih.gov/22677128/ 2. https://pubmed.ncbi.nlm.nih.gov/36849436/ 3. https://www.proteinatlas.org/ENSG00000133026‐MYH10/tissue

## 3 DISCUSSION

The association of denervation with clinically relevant indices of strength, physical performance, and muscle size with aging is poorly studied. To address this issue, we examined denervation‐responsive transcript abundance using whole transcriptomic analyses of muscle biopsies from 575 healthy men and women ≥70‐year‐old in the SOMMA cohort and used sensitivity analyses to address how this associated with SOMMA participant traits that included walking speed, VO_2peak_, maximal mitochondrial respiration, muscle strength, muscle power, D3Cr total body muscle mass, and total thigh muscle volume. We hypothesized that greater abundance of denervation‐responsive transcripts would associate positively with aging, but negatively with traits such as walking speed. Consistent with our hypothesis, higher expression of 13 denervation‐responsive genes associated positively with increasing age. Furthermore, elevated expression of denervation‐responsive genes was negatively associated with various functional and muscle size indices. For example, 400 m walking speed was negatively associated with increased transcript abundance for genes encoding a calcium‐dependent intercellular adhesion glycoprotein (CDH15), AChR subunits (CHRNA1, CHRND), denervation atrophy (GADD45A), and a transcription factor regulating aspects of muscle organization (RUNX1). Maximal mitochondrial respiration was negatively associated with increased transcript abundance for genes encoding CDH15, CHRNA1, CHRND, a glycoprotein that helps facilitate reinnervation (NCAM1), RUNX1, and a sodium channel (SCN5A). Similarly, leg power was negatively associated with elevated expression of genes encoding CDH15, CHRNA1, CHRND, another AChR subunit (CHRNE), NCAM1, RUNX1, and SCN5A. Collectively, our results are consistent with denervation playing an important role in driving declines in mobility and skeletal muscle function with aging in humans. In doing so, our findings underscore the importance of identifying strategies to maintain innervation of muscle fibers with aging.

Since the pioneering work of the Eccles which showed that switching the nerve supply between prototypical fast twitch versus slow twitch muscles made the fast muscles slower and the slow muscles faster (Buller et al., 1960), there has been an abundance of studies demonstrating the influence of muscle fiber innervation and muscle stimulation patterning on muscle phenotypes (Abruzzo et al., 2010; Ausoni et al., 1990; Patterson et al., 2006). It is thus not surprising that denervation of muscle fibers with aging is thought to play a very important role in driving hallmark features of aging muscle, including atrophy and fiber type shift (Hepple & Rice, 2016). Although impaired neuromuscular junction transmission is considered a plausible contributor to impaired muscle function in aging humans (Arnold & Clark, 2023), our understanding of how denervation relates to clinically important changes with aging, such as walking speed, is poorly studied. What we do know comes largely from smaller scale studies. For example, Piasecki and colleagues showed previously that older men with maintained muscle cross‐sectional area had larger motor unit potentials than younger men, whereas older men with low muscle cross‐sectional area did not. This finding was interpreted to indicate that the ability to reinnervate denervated muscle fibers, and thereby expand surviving motor units to compensate for those lost with aging, was essential to prevent muscle loss with aging (Piasecki et al., 2018). Similarly, our group have previously shown that motor unit number estimates are higher (Power et al., 2016), and indices of denervation lower (Sonjak, Jacob, Morais, et al., 2019), in world class octogenarian track and field athletes. While these studies are generally supportive of an important role for denervation in muscle mass and mobility with aging, there is a clear need for additional study using a larger number of participants representing a range of age and function. In this respect, the SOMMA is a large cohort study whose primary objective is to address how skeletal muscle biology relates to mobility in 879 healthy men and women between the ages of 70 and 94 (Cummings et al., 2023).

In the current study we mined whole transcriptome RNAseq data from vastus lateralis muscle biopsies of 575 men and women (≥70‐year‐old) who are SOMMA participants to determine how denervation‐responsive genes associate with clinically important physical traits, such as walking speed. In this regard, there are many studies in the literature which have used rodent models to identify sets of genes that change in response to experimental denervation (e.g., sciatic nerve transection) (Macpherson et al., 2015; Mugahid et al., 2019; Soares et al., 2014), including recent work that takes this down to single nucleus resolution (Lin et al., 2022). In selecting the denervation‐responsive genes for analysis in our current manuscript, we consulted these prior works, as well as reviews that address key components and signals involved in the mammalian neuromuscular junction (e.g., the agrin‐MuSK pathway, AChR subunits, etc.) (Rudolf et al., 2014; Tintignac et al., 2015), and a prior study that specifically addressed the impact of aging on the skeletal muscle transcriptome where denervation genes were highlighted amongst those with the most significant changes with aging (Ibebunjo et al., 2013). The selected genes encode proteins that are primarily found enriched in the muscle fiber side of the neuromuscular junction. A few others are preferentially expressed in activated muscle precursor/satellite cells (e.g., CDK5R1 (Lindqvist et al., 2017)) and injured perisynaptic Schwann cells (e.g., EFNA2 (Toma et al., 2020), SPOCK1 (Cifuentes‐Diaz et al., 2000)). Although it is clear that the severity/extent of muscle fiber denervation in aging muscle will be far less than in rodent surgical denervation models, the latter can provide a guide to which genes change in response to denervation. In this respect, based upon the abundance of small angular fibers that are typical of long‐term/persistent denervation (Dow et al., 2005) that we have characterized previously in ≥75‐year‐old men (Spendiff et al., 2016) and women (Sonjak, Jacob, Morais, et al., 2019), we estimate that our participants (mean age of 76 years at the time of study) would have 10%–20% persistently denervated muscle fibers within the biopsied material. We presume that the denervation‐responsive transcripts that we have characterized originate from these denervated muscle fibers.

It is important to note that several of the 49 denervation‐responsive genes that we selected are also known to change in other contexts. As such, although all 49 of the genes selected are well‐established as being responsive to denervation, there could be other processes beyond denervation influencing the expression of a given gene in aging muscle. It is likely that this pleiotropy of gene function and context contributes to some of the positive associations seen between select denervation‐responsive genes and SOMMA traits. Furthermore, it is also possible that the ability to upregulate expression of some of these denervation‐responsive genes could be related to positive outcomes because they promote successful reinnervation. Thus, increased expression of genes such as UTRN, APP, F2R and SPOCK1, which were positively associated with various SOMMA traits, may be indicators of successful reinnervation or other beneficial process in aging skeletal muscle rather than indicators of the burden of denervation per se. This issue is clearly worthy of further study.

Notwithstanding the point above about some genes having functions beyond denervation, consistent with our hypothesis, we observed that increased expression of many of the selected denervation‐responsive genes were negatively associated with clinically relevant measures in SOMMA participants. The clinical measures associated with the most denervation‐responsive genes were VO_2peak_ (15 genes) and maximal mitochondrial respiration (19 genes), where 12 of the genes were common between these traits. The high degree of correspondence between VO_2peak_ and maximal mitochondrial respiration is not unexpected given the association of these traits in the SOMMA cohort (Mau et al., 2022) and in the Baltimore Longitudinal Study of Aging (Gonzalez‐Freire et al., 2018). What may be more surprising is that maximal mitochondrial respiration was associated with transcriptomic indices of denervation burden. In this respect, denervation has been shown to induce the release of fatty acid hydroperoxides (a form of reactive oxygen species [ROS]) from the mitochondrial outer membrane and this is catalyzed by the enzyme cytoplasmic phospholipase A2 (cPLA2) (Bhattacharya et al., 2009). Furthermore, pharmacological inhibition of cPLA2 using an agent called arachidonyl trifluoromethyl ketone (AACOCF3) normalized mitochondrial ROS from denervated muscle (Bhattacharya et al., 2009). Our group has used this strategy in aging humans and found that at ages associated with muscle morphological and transcriptional changes indicative of denervation cPLA2 inhibition using AACOCF3 reduced ROS emission in skeletal muscle mitochondria from elderly men (Spendiff et al., 2016) and women (Sonjak, Jacob, Spendiff, et al., 2019). This finding suggests that denervation is amongst the factors contributing to mitochondrial changes in aging skeletal muscle. Further to the influence of denervation on mitochondria, there is also a marked decrease in mitochondrial enzymes with denervation (Abruzzo et al., 2010) secondary to the removal of mitochondria through mitophagy (Triolo et al., 2022). As such, denervation is well‐known to influence mitochondrial function and has also been implicated in altering some facets of mitochondrial function in aging human skeletal muscle previously. Our current findings add to this prior data in supporting the influence of denervation on mitochondrial function changes with aging.

Amongst the genes for which we observed significant negative associations with SOMMA traits was the transcription factor RUNX1. Interestingly, RUNX1 has been shown to be elevated in skeletal muscle of older patients with osteoporotic hip fracture and low muscle mass (Kang et al., 2021). Not only is RUNX1 elevated with denervation, but there is also a report observing a reduction in RUNX1 following resistance training in obese elderly participants and this was coincident with a reduction in the expression of the denervationresponsive glycoprotein NCAM1 at both the transcript level and protein level (NCAM protein) (Messi et al., 2016). The function of RUNX1 in the context of denervation was first described by Wang and colleagues where they showed that genetic knockout of RUNX1 led to an exacerbation of muscle impairment in the context of experimental denervation (Wang et al., 2005). They also identified that RUNX1 regulated the transcription of 29 genes that included ion channels, structural proteins, and signaling molecules. In this respect, a recent study identified RUNX1 as one of the transcriptions factors that can regulate SCN5A expression (Carreras et al., 2021), noting that SCN5A was amongst the denervation‐responsive genes that exhibited significant negative associations with VO_2peak_, maximal mitochondrial respiration, and leg power. Additional analyses of transcription factors related to the denervation‐responsive genes in our dataset would be of interest in a future investigation.

## 4 CONCLUSIONS

Our analysis of a set of denervation‐responsive genes in 575 participants in SOMMA has revealed significant associations between increased expression of denervation‐responsive genes and a diverse range of traits that include walking speed, VO_2peak_, maximal mitochondrial respiration, total thigh muscle volume, leg power, and leg strength. Our results support and add to the evidence that denervation plays an important role in driving declines in various indices of function that contribute to the decline in mobility with aging.

## 5 METHODS

### 5.1 Study population

The SOMMA is a prospective cohort study of mobility in community‐dwelling older adults. Participants for the current study were from the baseline cohort, enrolled between April 2019 and December 2021. SOMMA was conducted at two clinical sites: University of Pittsburgh (Pittsburgh, PA) and Wake Forest University School of Medicine (WinstonSalem, NC). Eligible participants were ≥70 years old at enrollment, had a body mass index (BMI) of 18–40 kg/m^2^, and were eligible for magnetic resonance (MR) imaging and a muscle tissue biopsy (Cummings et al., 2023). Individuals with specific implants or on anticoagulation therapy were excluded due to ineligibility for MR imaging and muscle tissue biopsy, respectively. Individuals were further excluded if they had active cancer or were in the advanced stages of heart failure, renal failure on dialysis, dementia, or Parkinson's disease. Participants must have been able to complete the 400 m walk; those who appeared as they might not be able to complete the 400 m walk at the in‐person screening visit completed a short distance walk (4 m) to ensure their walking speed as ≥0.6m/s.

Transportation to and from the clinic was provided to participants if needed for all study visits. The study protocol was approved by the Western Institutional Review Board Copernicus Group (WCG IRB; study number 20180764) and all participants provided written informed consent.

More information about SOMMA recruitment and study protocols can be found elsewhere (Cummings et al., 2023). In brief, baseline testing occurred across 3 clinic visits that were completed up to within 8 weeks of each other. The average time between Day 1 and Day 3 testing was 42 days (6 week). Day 1 included general clinic assessments (e.g., physical tests; 5 h), Day 2 included MR imaging and Cardiopulmonary Exercise Testing (MR and CPET, 2–3 h), and Day 3 included fasting specimen and tissue collection (2 h). There were 879 participants who completed Day 1 of baseline testing and had at least one primary SOMMA measure: CPET, MR imaging, or muscle tissue biopsy.

### 5.2 Study measures

#### 5.2.1 Cardiorespiratory fitness (VO2 peak)

Cardiorespiratory fitness was measured using gold‐standard VO_2_ peak from Cardiopulmonary Exercise Testing (CPET). A standardized CPET, using a modified Balke or manual protocol, was administered to participants to measure ventilatory gases, oxygen and carbon dioxide inhaled and exhaled during exercise (Balady et al., 2010). Participants who were excluded from the maximal effort symptom‐limited peak test had acute electrocardiogram (ECG) abnormalities, uncontrolled blood pressure or history of myocardial infarction, unstable angina or angioplasty in the preceding 6 months. Testing for VO_2_ peak began at the participant's preferred walking speed with incremental rate (0.5 mph) and/or slope (2.5%) increased in 2‐min stages until respiratory exchange ratio, ratio between VCO_2_ and VO_2_, was ≥1.05 and self‐reported Borg Rating of Perceived Exertion (REF) was ≥17. Blood pressure, pulse oximetry, and ECG were monitored throughout exercise. VO_2_ peak was determined in the BREEZESUITE software (MGC Diagnostics, St. Paul, MN) as the highest 30‐second average of VO_2_ (mL/min) achieved. The data were manually reviewed to ensure the VO_2_ peak was selected correctly for each participant.

### 5.3 Other measures

*P*articipants are asked to walk at their usual pace for 400 m from which walking speed was calculated. Whole‐body D3Cr muscle mass was measured in participants using a D3creatine dilution protocol as previously described (Cawthon et al., 2018). Knee extensor leg power was assessed using a Keiser Air 420 exercise machine in the same leg as the muscle biopsy. Resistance to test power was based on determination of the 1 repetition maximum leg extensor strength. Body mass was assessed by balance beam or digital scales and height by wall‐mounted stadiometers. An approximately 6‐min long MR scan was taken of the whole body to assess body composition including thigh muscle volume with image processing by AMRA Medical (Linge et al., 2018). The CHAMPS questionnaire (Stewart et al., 2001) was used to assess specific types and the context of physical activities.

### 5.4 Sample preparation and sequencing

#### 5.4.1 Skeletal muscle biopsy collection and processing

Percutaneous biopsies were collected from the middle region of the musculus vastus lateralis under local anesthesia using a Bergstrom canula with suction (Mau et al., 2022). Following this, the specimen was blotted dry of blood and interstitial fluid and dissected free of any connective tissue and intermuscular fat. Approximately 20 mg of the biopsy specimen was placed into ice‐cold BIOPS media (10 mM Ca–EGTA buffer, 0.1 M free calcium, 20 mM imidazole, 20 mM taurine, 50 mM potassium 2‐[N‐morpholino]‐ethanesulfonic acid, 0.5 mM dithiothreitol, 6.56 mM MgCl2, 5.77 mM ATP, and 15 mM phosphocreatine [PCr], pH 7.1) for respirometry, as previously described (22). Myofiber bundles of approximately 2–3 mg were teased apart using a pair of sharp tweezers and a small Petri dish containing ice‐cold BIOPS media. After mechanical preparation, myofiber bundles were chemically permeabilized for 30 min with saponin (2 mL of 50 μg/mL saponin in ice‐cold BIOPS solution) placed on ice and a rocker (25 rpm). Myofiber bundles were washed twice (10 min each) with ice‐cold MiR05 media (0.5 mM ethylenediaminetetraacetic acid, 3 mM MgCl2·6H2O, 60 mM K‐lactobionate, 20 mM taurine, 10 mM KH2PO4, 20 mM N‐2‐hydroxyethylpiperazine‐N′‐2‐ethanesulfonic acid, 110 mM sucrose, and 1 g/L bovine serum albumin, pH 7.1) on an orbital shaker (25 rpm). The second wash in MiR05 contained blebbistatin (25 μM), a myosin II ATPase inhibitor, that was used to inhibit muscle contraction. Fiber bundle wet weight was determined immediately after permeabilization using an analytical balance (Mettler Toledo, Columbus, OH).

#### 5.4.2 RNA library preparation and sequencing

Total RNA from frozen human skeletal muscle samples (~5 to 30mg) was prepared using Trizol solution (Invitrogen) according to the manufacturer's direction in 2.0mL Eppendorf safe‐lock tubes. Homogenization was performed using the Bullet Blender (NextAdvance, Raymertown NY USA) with an appropriate amount of stainless steel beads (autoclaved, 0.5~2mm, NextAdvance, Raymertown NY USA) at 4°C on Setting 8 (Max is 12) in 30 s bouts. The homogenization step was repeated five times for a total of 3 min with at least 1 min break between each bout. The removal of residual genomic DNA was performed by incubating the RNA sample with DNase (AM1907, Thermosci) plus RiboLock RNase inhibitor (EO0381, Thermisci) at 37°C for 25min in a heating block (400rpm). Cleanup of the RNA samples was done using the DNase inactivation reagent following instructions provided instructed by the manufacturer (AM1907, Thermosci). The RNA concentration and integrity were determined by using ThermoSci Nanodrop and Agilent Tapestation, where the average RNA Integrity Number (RIN) in our samples was 7.88.

To prepare RNAseq library, polyA mRNA was isolated from about 250ng total RNA by using NEBNext Poly(A) mRNA magnetic isolation module (E7490L, NEB) and mRNA library constructed by using NEBNext Ultra II directional RNA library Pre Kit for Illumina (E7760L, NEB). Equal molarity of RNAseq libraries were pooled and sequenced on Illumina NovaSeq (2X150bp) to reach 80M reads per sample.

### 5.5 Alignment and quality control

The reads from RNA‐sequencing were aligned to the Genome Reference Consortium Human Build 38 (GRCh38) using the HISAT2 software (Kim et al., 2019). Duplicated aligned reads were further marked and excluded using the Picardtools software. Expression count data were obtained using the HTseq software (PMID 25260700). Genes with a total count of ≤ 20 across all samples were filtered out to remove non‐expressed genes.

### 5.6 Pathway identification

For the analyses performed in this manuscript, we manually curated a list of 49 genes whose expression is altered in surgical denervation studies (Ebert et al., 2012; Macpherson et al., 2015; Mugahid et al., 2019; Soares et al., 2014) and which have previously been shown to be enriched amongst the differentially expressed genes seen in aging rat skeletal muscle (Ibebunjo et al., 2013). These include genes involved in maintenance of the AChR cluster (AGRN, LRP4, MUSK, RPSN), genes encoding the 5 AChR subunits (CHRNA, CHRNB, CHRND, CHRNE, CHRNG), genes promoting reinnervation (NCAM1, NRG1), genes encoding different isoforms of sodium channels (SCN4A, SCN5A), genes involved in the denervation atrophy pathway (MYOG, GADD45A), and other genes that typically exhibit increased expression with denervation.

### 5.7 Regression models and visualization

Expression levels of 49 denervation‐responsive genes were analyzed. Gene expression associations with traits were identified using negative binomial regression models as implemented by DESeq2 (Love et al., 2014) in R and adjusted for age, gender, clinic site, race/ethnicity, height, weight, hours per week in all exercise‐related activities (CHAMPS), multimorbidity count category, and sequencing batch. DESeq2 uses a negative binomial generalized linear model for differential analysis and applies the Wald test for the significance of GLM coefficients. The Benjamini‐Hochberg false discovery rate method was used for P‐value adjustment. Genes were considered differentially expressed according to the significance criteria of FDR <0.05. In negative binomial models, traits were modeled using the number of standard deviations (SDs) from each trait's mean.

Consequently, the reported log base 2‐fold changes reflect the change in gene expression per one SD unit increase in each trait. Volcano plots were created to visualize the differential expression of RNAs (ENSGs) associated with functional measures. Heat maps were created to summarize significant ENSG associations across all analyzed traits.

## AUTHOR CONTRIBUTIONS

SC, AN, SK, RTH, PMCa, KE and DE designed the study. XZ, KW, CW performed RNA sample preparation. HB, ZH, DE, KAE, CL, RTH, GT conducted the main analyses and drafted the manuscript. KAE, ZH, DE, HB conducted the genetic analyses. RTH, KE, DE, ZH, NEL contributed to the interpretation of the results. KAE, ZH contributed to data collection. RTH, SC, PMCa, AN, NEL, SK, PMCo critically revised the manuscript. All authors reviewed and approved the final version of the manuscript and KE, ZH, and HB had full access to the data in the study and accept responsibility to submit for publication.

## FUNDING INFORMATION

SOMMA is funded by the National Institute on Aging (NIA) grant number. R01AG059416. Study infrastructure support was funded in part by NIA Claude D. Pepper Older. American Independence Centers at University of Pittsburgh (P30AG024827) and Wake Forest. University (P30AG021332) and the Clinical and Translational Science Institutes, funded by the National Center for Advancing Translational Science at Wake Forest University (UL10TR001420).

## CONFLICT OF INTEREST STATEMENT

S.R.C. is a consultant to Bioage Labs. P.M.Ca. is a consultant to and owns stock in MyoCorps. All other authors declare no conflict of interest.

## Supporting information


Table S1.

## Data Availability

All SOMMA data are publicly available via a web portal. Updated datasets are released approximately every 6 months https://sommaonline.ucsf.edu/.
