# Correction to “Higher expression of denervation‐responsive genes is negatively associated with muscle volume and performance traits in the study of muscle, mobility, and aging (SOMMA)”

**DOI:** 10.1111/acel.14415

**Published:** 2024-11-19

Lukasiewicz CJ, Tranah GJ, Evans DS, et al. Higher expression of denervation‐responsive genes is negatively associated with muscle volume and performance traits in the study of muscle, mobility, and aging (SOMMA). *Aging Cell*. 2024;23(6):e14115. https://doi.org/10.1111/acel.14115

In Table 1 of the publication, the “Total Summary” value for 400 m walk speed is reported as 1.9 ± 0.2; however, it should be 1.1 ± 0.2.

We apologize for this error.

