# Supplementary material for: Higher expression of denervation‐responsive genes is negatively associated with muscle volume and performance traits in the study of muscle, mobility, and aging (SOMMA)
Source: Aging Cell. 2024 Jun 3;23(6):e14115. doi: 10.1111/acel.14115 (PMC11166368; doi:10.1111/acel.14115)
Supplement: Supplementary file 1 — Table S1. [file ACEL-23-e14115-s001.pdf]

| Sample | Number of raw pairs | Alignment Rate | Number of aligned reads | Duplication Rate | Number of aligned reads deduplicated |
|---|---|---|---|---|---|
| 1 | 96393453 | 95.67% | 184441951 | 78.70% | 39359245 |
| 2 | 100059418 | 96.13% | 192367958 | 68.10% | 61433169 |
| 3 | 93532566 | 96.08% | 179727375 | 68.50% | 56555649 |
| 4 | 90130020 | 95.93% | 172917121 | 62.90% | 64215917 |
| 5 | 93513176 | 95.74% | 179061926 | 66.50% | 60023883 |
| 6 | 93399507 | 96.03% | 179390873 | 78.60% | 38357663 |
| 7 | 91533891 | 96.04% | 175814274 | 75.50% | 43079694 |
| 8 | 95660352 | 95.15% | 182046440 | 86% | 25426123 |
| 9 | 92869196 | 95.60% | 177559973 | 73.80% | 46546318 |
| 10 | 94206508 | 95.61% | 180140177 | 65.40% | 62368154 |
| 11 | 91580510 | 96.24% | 176269851 | 68.30% | 55963786 |
| 12 | 96476311 | 96.31% | 185835269 | 65.40% | 64275793 |
| 13 | 93256753 | 96.06% | 179170646 | 69.90% | 53926180 |
| 14 | 94737694 | 96.51% | 182871302 | 70.80% | 53335997 |
| 15 | 94853873 | 94.61% | 179484404 | 91.60% | 15094298 |
| 16 | 93254346 | 96.10% | 179236781 | 79.80% | 36214863 |
| 17 | 94768451 | 96.06% | 182078220 | 70.30% | 53994168 |
| 18 | 92703785 | 96.36% | 178650402 | 61.20% | 69327789 |
| 19 | 92792742 | 96.33% | 178781101 | 65.80% | 61190811 |
| 20 | 95448973 | 96.50% | 184214920 | 71.60% | 52242426 |
| 21 | 96386549 | 95.70% | 184490763 | 65.20% | 64208390 |
| 22 | 94049712 | 95.77% | 180144045 | 85.40% | 26374250 |
| 23 | 92534259 | 95.52% | 176781852 | 82% | 31751068 |
| 24 | 96136369 | 95.47% | 183567705 | 77.70% | 40894545 |
| 25 | 92684795 | 96.66% | 179169760 | 79% | 37553138 |
| 26 | 94274385 | 95.62% | 180291886 | 81% | 34329087 |
| 27 | 95238852 | 95.32% | 181565967 | 81.80% | 32954237 |
| 28 | 94658476 | 95.26% | 180343006 | 89.90% | 18235077 |
| 29 | 92917699 | 95.43% | 177341522 | 75.20% | 43975639 |
| 30 | 97826542 | 96.21% | 188243115 | 72.50% | 51785757 |
| 31 | 93211235 | 95.45% | 177942884 | 83.40% | 29533846 |
| 32 | 95606463 | 95.69% | 182973610 | 75.20% | 45433035 |
| 33 | 95441961 | 96.31% | 183849801 | 74.60% | 46701458 |
| 34 | 96075319 | 96.12% | 184697195 | 74.20% | 47673981 |
| 35 | 95074060 | 96.35% | 183208134 | 70.80% | 53417857 |
| 36 | 91693188 | 95.82% | 175727627 | 77.60% | 39393394 |
| 37 | 92617017 | 92.67% | 171647448 | 90.10% | 16974404 |
| 38 | 95760744 | 95.74% | 183363658 | 78% | 40363306 |
| 39 | 93959617 | 94.95% | 178419945 | 82.10% | 32008520 |
| 40 | 97687230 | 95.69% | 186945742 | 72.80% | 50891281 |
| 41 | 95290985 | 96.08% | 183118608 | 76.10% | 43755133 |
| 42 | 96405400 | 96.10% | 185288591 | 71.30% | 53153408 |
| 43 | 91618248 | 95.70% | 175361499 | 74% | 45638953 |
| 44 | 90604635 | 95.59% | 173221766 | 79.70% | 35235993 |
| 45 | 91908525 | 94.24% | 173221121 | 73.90% | 45147052 |
| 46 | 93339668 | 95.45% | 178183219 | 86.60% | 23847280 |
| 47 | 93228195 | 95.38% | 177838787 | 80.10% | 35379447 |
| 48 | 94185936 | 95.70% | 180277596 | 85.90% | 25478974 |
| 49 | 93076777 | 95.60% | 177965955 | 74.90% | 44623024 |
| 50 | 92853231 | 95.51% | 177372625 | 67.50% | 57638727 |
| 51 | 95924802 | 95.83% | 183844971 | 83.20% | 30890475 |
| 52 | 91701902 | 93.61% | 171676642 | 88.50% | 19794352 |
| 53 | 93978044 | 95.60% | 179680494 | 82.20% | 31952553 |
| 54 | 93498088 | 94.84% | 177339169 | 85.90% | 25052790 |
| 55 | 91373701 | 95.10% | 173792287 | 86% | 24339052 |
| 56 | 92633161 | 94.21% | 174537705 | 92.40% | 13282764 |
| 57 | 93632029 | 94.82% | 177555337 | 74.70% | 44895907 |
| 58 | 96935810 | 96.11% | 186322556 | 66.40% | 62655363 |
| 59 | 95970326 | 95.97% | 184214134 | 68.70% | 57608266 |
| 60 | 92800167 | 95.46% | 177179393 | 86.60% | 23666468 |
| 61 | 102699034 | 96.30% | 197801041 | 64.90% | 69504546 |
| 62 | 92654946 | 94.91% | 175870882 | 85% | 26296527 |

| | | | | | |
|---|---|---|---|---|---|
| 63 | 92637261 | 94.25% | 174620099 | 85.40% | 25547736 |
| 64 | 93933534 | 94.91% | 178301144 | 86% | 24893302 |
| 65 | 94850969 | 94.69% | 179622664 | 91.10% | 15967433 |
| 66 | 93576231 | 95.41% | 178562087 | 80.90% | 34065450 |
| 67 | 90485671 | 95.26% | 172384541 | 75.90% | 41587116 |
| 68 | 93379297 | 94.19% | 175909909 | 91.20% | 15397217 |
| 69 | 96498988 | 95.91% | 185101771 | 73.10% | 49753846 |
| 70 | 71461019 | 95.40% | 136344256 | 59.30% | 55504737 |
| 71 | 93752448 | 95.60% | 179248936 | 59.20% | 73073568 |
| 72 | 86411126 | 96.23% | 166306753 | 58.30% | 69376925 |
| 73 | 83235259 | 96.26% | 160250568 | 55.50% | 71262583 |
| 74 | 81261596 | 96.36% | 156615091 | 60.10% | 62471112 |
| 75 | 97783727 | 95.85% | 187450816 | 57.10% | 80348879 |
| 76 | 83767720 | 95.75% | 160407526 | 57.60% | 68092398 |
| 77 | 86729806 | 95.31% | 165318863 | 57.60% | 70036456 |
| 78 | 110942864 | 95.51% | 211919823 | 57% | 91162871 |
| 79 | 104966827 | 96.19% | 201945250 | 56% | 88772846 |
| 80 | 98253075 | 96.48% | 189598150 | 65.60% | 65198970 |
| 81 | 116927639 | 96.27% | 225129926 | 56.50% | 97959452 |
| 82 | 112176949 | 96.19% | 215797160 | 58.40% | 89813138 |
| 83 | 98534039 | 95.80% | 188796585 | 53.50% | 87847469 |
| 84 | 108954720 | 95.86% | 208892196 | 53.40% | 97416327 |
| 85 | 84097255 | 95.78% | 161090321 | 57.10% | 69129756 |
| 86 | 95236027 | 95.90% | 182663085 | 61.60% | 70206810 |
| 87 | 88597819 | 95.87% | 169884224 | 55.30% | 75955704 |
| 88 | 96328455 | 96.12% | 185180033 | 57.80% | 78177200 |
| 89 | 90461109 | 96.09% | 173855662 | 56.80% | 75088574 |
| 90 | 102870253 | 95.60% | 196695477 | 57.10% | 84377364 |
| 91 | 108655599 | 96.07% | 208762677 | 55% | 93925156 |
| 92 | 106334623 | 95.39% | 202873100 | 58% | 85169516 |
| 93 | 95273543 | 96.52% | 183917484 | 59.50% | 74534598 |
| 94 | 92910303 | 95.10% | 176722146 | 61.70% | 67734224 |
| 95 | 85877520 | 95.74% | 164431755 | 57.30% | 70181106 |
| 96 | 101420521 | 96.01% | 194746668 | 57.80% | 82270980 |
| 97 | 106560498 | 95.79% | 204151071 | 60.10% | 81465013 |
| 98 | 88052217 | 95.85% | 168790927 | 61.40% | 65182021 |
| 99 | 86639281 | 95.98% | 166305148 | 60.20% | 66160365 |
| 100 | 110166267 | 96.12% | 211781602 | 58.40% | 88101366 |
| 101 | 68517291 | 95.90% | 131415364 | 58.40% | 54667509 |
| 102 | 82185872 | 89.49% | 147099825 | 56.90% | 63463056 |
| 103 | 81881951 | 91.49% | 149826924 | 51.60% | 72577249 |
| 104 | 79982310 | 92.80% | 148454721 | 54.20% | 68030223 |
| 105 | 80404905 | 93.43% | 150237431 | 54.10% | 68971717 |
| 106 | 80821283 | 92.89% | 150151720 | 56.80% | 64805299 |
| 107 | 81203942 | 92.14% | 149636180 | 52.50% | 71064306 |
| 108 | 83124572 | 93.65% | 155692925 | 55% | 70035174 |
| 109 | 83732758 | 91.81% | 153750133 | 56% | 67697013 |
| 110 | 85425657 | 93.21% | 159252490 | 55.20% | 71368045 |
| 111 | 83608132 | 92.59% | 154821249 | 55.10% | 69477695 |
| 112 | 80057039 | 92.61% | 148280864 | 51.40% | 72014599 |
| 113 | 80421460 | 92.78% | 149231205 | 55.70% | 66145867 |
| 114 | 79661371 | 93.39% | 148792790 | 52.40% | 70837393 |
| 115 | 78552545 | 93.67% | 147161472 | 49.60% | 74116342 |
| 116 | 80742166 | 93.70% | 151310619 | 53.80% | 69932072 |
| 117 | 81952954 | 93.45% | 153170108 | 51.40% | 74467136 |
| 118 | 84605721 | 92.48% | 156488222 | 53.50% | 72754366 |
| 119 | 83317876 | 93.43% | 155693857 | 54.60% | 70715421 |
| 120 | 84072070 | 92.31% | 155213840 | 55.10% | 69658730 |
| 121 | 78879445 | 93.85% | 148060431 | 54.30% | 67613149 |
| 122 | 81065245 | 93.13% | 150987465 | 52.90% | 71065006 |
| 123 | 80217494 | 93.74% | 150386891 | 52.60% | 71283062 |
| 124 | 78825058 | 93.19% | 146907604 | 53% | 69007099 |
| 125 | 82173370 | 92.22% | 151561681 | 56% | 66722801 |

| | | | | | |
|---|---|---|---|---|---|
| 126 | 84325245 | 91.89% | 154967259 | 55.80% | 68529272 |
| 127 | 83005575 | 92.12% | 152925352 | 52.40% | 72862136 |
| 128 | 79764300 | 92.28% | 147216468 | 54.60% | 66881432 |
| 129 | 80033284 | 93.50% | 149658311 | 53.60% | 69425984 |
| 130 | 83094845 | 92.07% | 153013625 | 56% | 67339202 |
| 131 | 81833515 | 93.24% | 152604746 | 53.20% | 71429012 |
| 132 | 81767329 | 92.75% | 151672136 | 54.50% | 69022261 |
| 133 | 86613650 | 93.83% | 162536626 | 50.90% | 79868855 |
| 134 | 84575964 | 93.04% | 157374691 | 52.80% | 74277949 |
| 135 | 84551330 | 93.20% | 157608706 | 57.30% | 67364855 |
| 136 | 79377942 | 92.20% | 146379254 | 54.60% | 66496956 |
| 137 | 82220781 | 93.55% | 153841128 | 53.80% | 71087389 |
| 138 | 84490367 | 93.66% | 158262891 | 49% | 80737589 |
| 139 | 79135730 | 93.46% | 147925972 | 54.60% | 67200337 |
| 140 | 79315800 | 93.29% | 147985482 | 53.80% | 68321650 |
| 141 | 83183251 | 93.78% | 156011569 | 56.20% | 68318618 |
| 142 | 80033861 | 93.20% | 149184350 | 54.50% | 67886689 |
| 143 | 81365287 | 93.54% | 152211890 | 53.10% | 71334270 |
| 144 | 78302039 | 93.65% | 146653385 | 52% | 70451521 |
| 145 | 78250641 | 93.72% | 146677528 | 50.20% | 72978169 |
| 146 | 88982141 | 93.56% | 166509772 | 51.30% | 81144529 |
| 147 | 83465583 | 93.76% | 156512508 | 52.70% | 73991949 |
| 148 | 78062842 | 93.16% | 145453918 | 54.90% | 65605230 |
| 149 | 82988051 | 93.61% | 155366184 | 54.10% | 71328165 |
| 150 | 81862895 | 93.18% | 152566757 | 53.50% | 70874912 |
| 151 | 83357795 | 92.99% | 155033190 | 53.90% | 71547631 |
| 152 | 78541474 | 92.61% | 145477010 | 55.20% | 65238613 |
| 153 | 79658492 | 93.21% | 148502581 | 54% | 68314118 |
| 154 | 83568623 | 93.12% | 155630779 | 51.10% | 76070555 |
| 155 | 82216893 | 93.36% | 153507981 | 54.80% | 69417332 |
| 156 | 80284841 | 93.01% | 149342427 | 53.80% | 69032431 |
| 157 | 81855521 | 92.63% | 151649262 | 57.70% | 64177810 |
| 158 | 83777511 | 92.15% | 154404604 | 56.40% | 67384639 |
| 159 | 76397124 | 92.84% | 141852133 | 52.10% | 67974888 |
| 160 | 76699266 | 93.64% | 143635884 | 52.50% | 68190049 |
| 161 | 81119697 | 92.98% | 150848135 | 57.30% | 64381281 |
| 162 | 80828343 | 93.88% | 151762556 | 53.70% | 70201572 |
| 163 | 81754170 | 92.54% | 151315363 | 54.40% | 68934509 |
| 164 | 77182270 | 92.88% | 143378696 | 54.50% | 65188366 |
| 165 | 83277951 | 91.93% | 153115251 | 54.80% | 69242593 |
| 166 | 81434647 | 93.24% | 151858802 | 53% | 71313059 |
| 167 | 80097377 | 93.07% | 149095520 | 56% | 65587909 |
| 168 | 80351222 | 92.75% | 149049068 | 55.40% | 66477867 |
| 169 | 81065470 | 92.60% | 150136734 | 56.50% | 65376792 |
| 170 | 79732292 | 92.77% | 147932802 | 51.20% | 72190979 |
| 171 | 81364923 | 92.82% | 151041094 | 54.70% | 68468523 |
| 172 | 84217555 | 92.71% | 156156243 | 53.40% | 72719070 |
| 173 | 82659700 | 91.86% | 151855951 | 58.90% | 62427158 |
| 174 | 77764144 | 92.67% | 144122794 | 54.70% | 65326768 |
| 175 | 78458276 | 93.61% | 146894807 | 53.40% | 68434532 |
| 176 | 82281245 | 92.59% | 152375583 | 54.70% | 69096970 |
| 177 | 81723750 | 93.04% | 152071478 | 53.70% | 70467804 |
| 178 | 92966002 | 93.08% | 173061954 | 53% | 81409313 |
| 179 | 108642167 | 92.90% | 201857878 | 54.50% | 91931884 |
| 180 | 110042963 | 93.11% | 204915230 | 55.40% | 91326091 |
| 181 | 80782419 | 91.74% | 148214005 | 55.40% | 66133720 |
| 182 | 84525342 | 93.26% | 157656520 | 54.20% | 72184934 |
| 183 | 83707351 | 92.93% | 155580510 | 56.70% | 67374897 |
| 184 | 81396801 | 92.67% | 150863483 | 54.20% | 69072120 |
| 185 | 79461855 | 92.93% | 147683370 | 53.60% | 68516893 |
| 186 | 82835786 | 93.45% | 154824888 | 56.10% | 68011860 |
| 187 | 80121046 | 91.52% | 146653254 | 56.20% | 64198018 |
| 188 | 80677720 | 93.08% | 150191167 | 51.70% | 72467988 |

| | | | | | |
|---|---|---|---|---|---|
| 189 | 73976840 | 92.95% | 137522860 | 55.50% | 61139754 |
| 190 | 84296590 | 92.68% | 156244470 | 54.30% | 71397717 |
| 191 | 79357053 | 92.05% | 146094379 | 57.40% | 62260719 |
| 192 | 83738743 | 92.78% | 155381196 | 55.10% | 69733453 |
| 193 | 81718359 | 93.00% | 151991385 | 55.70% | 67276304 |
| 194 | 82041039 | 92.73% | 152145911 | 53.70% | 70411580 |
| 195 | 80029302 | 93.27% | 149286808 | 54.60% | 67736131 |
| 196 | 80785074 | 92.28% | 149102807 | 58.20% | 62289713 |
| 197 | 83843652 | 89.95% | 150834458 | 55.60% | 66959638 |
| 198 | 83590981 | 90.54% | 151374766 | 54.40% | 69016209 |
| 199 | 83437723 | 90.56% | 151119766 | 61.30% | 58460828 |
| 200 | 83200552 | 90.82% | 151130394 | 58.30% | 63046406 |
| 201 | 85626471 | 91.26% | 156284569 | 54.70% | 70851462 |
| 202 | 85639782 | 91.15% | 156127532 | 55.30% | 69759580 |
| 203 | 82537377 | 92.16% | 152128868 | 54.80% | 68808968 |
| 204 | 85570850 | 91.07% | 155854052 | 55.50% | 69431392 |
| 205 | 85934669 | 88.37% | 151874286 | 57.70% | 64291224 |
| 206 | 82777122 | 89.44% | 148070369 | 56.30% | 64655527 |
| 207 | 84965709 | 91.77% | 155946997 | 60.90% | 60917009 |
| 208 | 83933360 | 91.66% | 153860969 | 56.30% | 67229061 |
| 209 | 84894553 | 91.66% | 155625165 | 54.60% | 70729417 |
| 210 | 86259859 | 91.23% | 157396509 | 57.40% | 67053274 |
| 211 | 84346300 | 90.21% | 152170674 | 60.30% | 60352643 |
| 212 | 81677653 | 92.44% | 151003784 | 54% | 69511530 |
| 213 | 84048781 | 90.99% | 152954587 | 54.50% | 69606002 |
| 214 | 83654135 | 91.72% | 153456971 | 56.10% | 67299283 |
| 215 | 82130420 | 91.59% | 150453661 | 55.80% | 66435733 |
| 216 | 86286063 | 92.62% | 159833996 | 55.20% | 71548012 |
| 217 | 81626143 | 92.37% | 150800166 | 53.50% | 70150436 |
| 218 | 88346127 | 92.33% | 163143773 | 61.10% | 63421969 |
| 219 | 84865918 | 91.48% | 155278413 | 59.90% | 62191416 |
| 220 | 84973197 | 91.01% | 154664003 | 57.10% | 66420352 |
| 221 | 85254795 | 91.64% | 156259490 | 52.70% | 73942216 |
| 222 | 83789654 | 91.01% | 152511134 | 58.80% | 62864169 |
| 223 | 95815599 | 92.47% | 177201844 | 57.80% | 74694621 |
| 224 | 81589169 | 92.29% | 150602813 | 53.40% | 70253955 |
| 225 | 84008407 | 93.45% | 157012490 | 54.40% | 71645670 |
| 226 | 91458190 | 92.84% | 169827777 | 52% | 81521417 |
| 227 | 85816384 | 92.18% | 158208155 | 51.90% | 76103627 |
| 228 | 85124387 | 92.51% | 157502606 | 53.30% | 73491302 |
| 229 | 95718199 | 92.66% | 177393846 | 50.90% | 87012401 |
| 230 | 88765558 | 91.84% | 163040208 | 50.20% | 81131345 |
| 231 | 85307518 | 92.10% | 157139563 | 47.20% | 82950928 |
| 232 | 85215525 | 92.17% | 157078878 | 52.20% | 75159281 |
| 233 | 92867670 | 92.78% | 172320565 | 50.30% | 85711818 |
| 234 | 95802997 | 92.10% | 176464214 | 51% | 86392608 |
| 235 | 90056750 | 92.42% | 166462816 | 52.40% | 79208945 |
| 236 | 88674254 | 92.26% | 163617746 | 49.10% | 83319295 |
| 237 | 87963713 | 93.34% | 164213747 | 49.40% | 83028930 |
| 238 | 92809866 | 92.70% | 172061848 | 51.70% | 83186393 |
| 239 | 87668758 | 92.36% | 161935944 | 54.80% | 73257571 |
| 240 | 83691132 | 93.13% | 155876722 | 52.30% | 74285916 |
| 241 | 87620635 | 93.43% | 163722214 | 48.50% | 84303328 |
| 242 | 87370476 | 93.83% | 163955893 | 51.50% | 79457633 |
| 243 | 85183296 | 93.51% | 159304394 | 48.90% | 81388632 |
| 244 | 85305143 | 91.00% | 155259108 | 59.40% | 62964001 |
| 245 | 87686269 | 91.76% | 160913835 | 50.10% | 80229213 |
| 246 | 85759147 | 92.88% | 159301455 | 45.80% | 86380616 |
| 247 | 86761827 | 92.46% | 160444212 | 52.30% | 76597750 |
| 248 | 95574789 | 93.14% | 178043997 | 53.60% | 82642295 |
| 249 | 84799240 | 91.03% | 154390548 | 58.90% | 63417450 |
| 250 | 92994203 | 92.25% | 171580708 | 54.90% | 77303002 |
| 251 | 90002275 | 93.51% | 168319214 | 52.50% | 79979804 |

| | | | | | |
|---|---|---|---|---|---|
| 252 | 93496332 | 92.41% | 172791290 | 53.20% | 80942510 |
| 253 | 93429096 | 93.13% | 174026090 | 53.40% | 81155282 |
| 254 | 92210444 | 93.87% | 173118774 | 49.60% | 87229532 |
| 255 | 86830309 | 93.01% | 161524479 | 51.20% | 78790992 |
| 256 | 85668489 | 93.69% | 160529297 | 53.20% | 75101647 |
| 257 | 82811822 | 93.50% | 154851807 | 56.70% | 67098167 |
| 258 | 92210607 | 92.75% | 171043151 | 56.20% | 74920206 |
| 259 | 86670973 | 92.98% | 161175596 | 54% | 74204742 |
| 260 | 85317863 | 92.59% | 157985436 | 53.60% | 73349237 |
| 261 | 84376356 | 93.29% | 157430753 | 52% | 75551090 |
| 262 | 88846406 | 93.69% | 166474759 | 52.80% | 78535808 |
| 263 | 84250864 | 93.37% | 157323970 | 49.90% | 78810698 |
| 264 | 83884503 | 93.41% | 156707758 | 55.70% | 69362822 |
| 265 | 84039086 | 93.25% | 156727952 | 58.10% | 65613249 |
| 266 | 85755986 | 93.72% | 160745223 | 52.40% | 76444502 |
| 267 | 84323917 | 93.02% | 156873725 | 55.10% | 70465299 |
| 268 | 86565553 | 93.53% | 161926567 | 50.80% | 79604802 |
| 269 | 86567025 | 93.16% | 161289477 | 50.10% | 80523325 |
| 270 | 88494339 | 92.87% | 164361568 | 52% | 78929966 |
| 271 | 86623009 | 93.02% | 161151152 | 51.20% | 78600588 |
| 272 | 86327648 | 93.10% | 160745980 | 54% | 73918147 |
| 273 | 83141346 | 93.24% | 155036034 | 53.30% | 72386235 |
| 274 | 83302444 | 93.24% | 155343331 | 60.30% | 61680278 |
| 275 | 84909067 | 93.47% | 158736296 | 53.10% | 74517144 |
| 276 | 94998934 | 93.04% | 176782669 | 54.20% | 80973713 |
| 277 | 85473106 | 91.72% | 156783969 | 48% | 81529587 |
| 278 | 84419283 | 93.08% | 157151408 | 49.60% | 79147797 |
| 279 | 84339155 | 92.54% | 156093345 | 54.10% | 71659275 |
| 280 | 82898656 | 93.49% | 155005772 | 57.10% | 66469529 |
| 281 | 88618338 | 92.36% | 163687680 | 55.50% | 72864712 |
| 282 | 84028068 | 92.83% | 155998753 | 55.40% | 69596791 |
| 283 | 86880065 | 92.54% | 160795831 | 54.20% | 73623117 |
| 284 | 84845899 | 93.53% | 158711551 | 51.20% | 77485314 |
| 285 | 84860629 | 92.11% | 156330197 | 52.40% | 74379075 |
| 286 | 84257108 | 93.42% | 157421881 | 52.40% | 74920353 |
| 287 | 84280613 | 93.66% | 157878588 | 50.10% | 78725756 |
| 288 | 84861735 | 93.52% | 158721939 | 53.70% | 73496627 |
| 289 | 85386584 | 93.20% | 159158664 | 51.50% | 77183783 |
| 290 | 85547278 | 91.97% | 157357004 | 64.40% | 56086510 |
| 291 | 85506856 | 92.17% | 157623630 | 50.60% | 77883014 |
| 292 | 87465435 | 92.87% | 162465151 | 48.70% | 83288893 |
| 293 | 89476865 | 93.44% | 167220449 | 50.30% | 83141110 |
| 294 | 82110594 | 93.53% | 153591414 | 55% | 69078281 |
| 295 | 84093778 | 92.59% | 155729679 | 60.40% | 61678538 |
| 296 | 83423489 | 93.01% | 155185118 | 59.60% | 62749001 |
| 297 | 85932540 | 93.29% | 160337553 | 59.40% | 65131305 |
| 298 | 83538416 | 93.34% | 155953809 | 52.40% | 74289150 |
| 299 | 84091188 | 93.52% | 157277665 | 49.20% | 79941873 |
| 300 | 90069946 | 93.01% | 167540972 | 52.10% | 80254592 |
| 301 | 84061111 | 93.66% | 157457891 | 50.80% | 77394403 |
| 302 | 83155272 | 93.16% | 154932286 | 57.50% | 65789921 |
| 303 | 83585064 | 92.55% | 154711276 | 56.30% | 67639225 |
| 304 | 84628574 | 93.46% | 158188844 | 55.90% | 69742868 |
| 305 | 83011782 | 92.14% | 152977493 | 54.20% | 70059215 |
| 306 | 81280441 | 93.78% | 152451484 | 50.80% | 74998130 |
| 307 | 84245261 | 92.76% | 156299058 | 54% | 71892451 |
| 308 | 84725802 | 92.59% | 156893654 | 51.20% | 76591677 |
| 309 | 84866329 | 92.66% | 157282114 | 52.60% | 74575212 |
| 310 | 85688224 | 92.84% | 159099875 | 53.30% | 74358069 |
| 311 | 85437828 | 91.87% | 156986962 | 62.70% | 58478149 |
| 312 | 84433153 | 92.86% | 156816220 | 55.70% | 69523283 |
| 313 | 84621049 | 92.92% | 157251935 | 53.20% | 73551058 |
| 314 | 83144014 | 93.91% | 156162315 | 50.30% | 77646238 |

| | | | | | |
|---|---|---|---|---|---|
| 315 | 83362769 | 92.86% | 154822328 | 50.70% | 76272766 |
| 316 | 84481479 | 93.31% | 157655895 | 49.30% | 79968258 |
| 317 | 86230336 | 93.73% | 161655502 | 50.80% | 79536622 |
| 318 | 84799431 | 92.73% | 157261019 | 49.80% | 78957526 |
| 319 | 78405835 | 93.18% | 146109354 | 48% | 75928263 |
| 320 | 88892787 | 93.76% | 166692480 | 50.80% | 82020430 |
| 321 | 85070862 | 93.32% | 158769321 | 48% | 82521937 |
| 322 | 84947408 | 94.30% | 160214849 | 50.80% | 78829492 |
| 323 | 92407695 | 93.73% | 173228551 | 52.50% | 82368708 |
| 324 | 84891874 | 94.27% | 160048590 | 50.70% | 78926705 |
| 325 | 85709910 | 94.39% | 161804370 | 51.50% | 78417315 |
| 326 | 83101247 | 94.01% | 156241366 | 48.50% | 80410232 |
| 327 | 82308793 | 93.72% | 154274395 | 52.10% | 73843575 |
| 328 | 87109225 | 94.00% | 163759092 | 51% | 80301894 |
| 329 | 86532923 | 92.20% | 159572561 | 50.90% | 78415267 |
| 330 | 90737882 | 90.54% | 164315402 | 54.90% | 74053890 |
| 331 | 83355319 | 92.29% | 153862208 | 50.30% | 76523033 |
| 332 | 85026830 | 93.60% | 159169119 | 51.60% | 76985647 |
| 333 | 83133743 | 92.89% | 154448461 | 52.50% | 73314153 |
| 334 | 81974796 | 92.52% | 151679531 | 49.70% | 76328624 |
| 335 | 85411349 | 93.16% | 159131845 | 48.70% | 81630732 |
| 336 | 87230169 | 89.93% | 156896035 | 51.20% | 76597809 |
| 337 | 86522843 | 93.28% | 161421146 | 52.10% | 77300193 |
| 338 | 83912636 | 92.91% | 155918473 | 47.40% | 81939343 |
| 339 | 96064117 | 93.21% | 179087734 | 55.50% | 79780353 |
| 340 | 91874480 | 92.19% | 169398960 | 54.10% | 77734137 |
| 341 | 90458184 | 93.45% | 169069924 | 54.20% | 77368105 |
| 342 | 92262874 | 92.32% | 170355177 | 56.30% | 74361329 |
| 343 | 88623713 | 93.33% | 165428691 | 54.40% | 75387321 |
| 344 | 87207805 | 91.69% | 159918728 | 62.10% | 60610291 |
| 345 | 91004676 | 91.07% | 165761485 | 65.50% | 57159741 |
| 346 | 90916275 | 93.80% | 170560323 | 54.10% | 78288436 |
| 347 | 82776921 | 91.55% | 151571920 | 65.70% | 52028736 |
| 348 | 92768155 | 91.99% | 170681073 | 52.80% | 80587319 |
| 349 | 87856604 | 91.93% | 161536091 | 56% | 71121274 |
| 350 | 94246257 | 93.71% | 176642932 | 54% | 81343271 |
| 351 | 90609020 | 92.28% | 167228668 | 60.30% | 66404884 |
| 352 | 96498298 | 92.76% | 179016244 | 60% | 71599729 |
| 353 | 90315456 | 92.62% | 167293252 | 57.70% | 70834030 |
| 354 | 91413142 | 91.00% | 166373604 | 59.60% | 67260982 |
| 355 | 87915585 | 92.42% | 162503027 | 61.70% | 62305118 |
| 356 | 91125683 | 92.91% | 169336075 | 57.40% | 72191861 |
| 357 | 91398473 | 92.76% | 169571520 | 54.20% | 77661393 |
| 358 | 95481994 | 91.81% | 175329119 | 60.40% | 69382289 |
| 359 | 110818214 | 92.60% | 205241457 | 57.60% | 87117111 |
| 360 | 94806068 | 92.95% | 176246636 | 54.70% | 79789029 |
| 361 | 89836893 | 92.71% | 166580194 | 56.10% | 73142972 |
| 362 | 98638711 | 93.23% | 183921845 | 56.70% | 79659069 |
| 363 | 93853377 | 92.12% | 172917042 | 55.50% | 77019448 |
| 364 | 100275484 | 91.79% | 184078267 | 58.40% | 76604986 |
| 365 | 92495429 | 93.01% | 172062371 | 54.80% | 77718704 |
| 366 | 88715899 | 92.84% | 164731494 | 55.50% | 73260860 |
| 367 | 92461693 | 93.31% | 172556715 | 55.80% | 76329898 |
| 368 | 102100512 | 92.74% | 189382528 | 54% | 87061978 |
| 369 | 90504044 | 92.70% | 167798855 | 60.10% | 66899554 |
| 370 | 89709289 | 93.94% | 168552896 | 52.20% | 80537509 |
| 371 | 87126691 | 93.41% | 162764208 | 60.10% | 64993578 |
| 372 | 84451594 | 93.81% | 158447618 | 54.30% | 72379951 |
| 373 | 92484999 | 93.30% | 172582788 | 56.20% | 75514636 |
| 374 | 95069991 | 92.37% | 175627025 | 53.60% | 81522149 |
| 375 | 91417795 | 93.66% | 171251489 | 54.30% | 78191020 |
| 376 | 87960991 | 90.90% | 159916106 | 59.70% | 64483465 |
| 377 | 99886475 | 93.44% | 186660828 | 57.40% | 79514036 |

| | | | | | |
|---|---|---|---|---|---|
| 378 | 87890978 | 92.47% | 162553511 | 57.30% | 69367802 |
| 379 | 92017579 | 93.07% | 171275765 | 61.20% | 66437963 |
| 380 | 87881414 | 93.31% | 163996843 | 54.10% | 75296785 |
| 381 | 88237604 | 93.83% | 165581376 | 52.10% | 79392676 |
| 382 | 87808127 | 93.01% | 163340130 | 55.70% | 72401527 |
| 383 | 91892021 | 94.02% | 172792672 | 52.20% | 82574586 |
| 384 | 86824993 | 93.68% | 162683119 | 53.70% | 75385841 |
| 385 | 83169708 | 92.44% | 153768393 | 64.50% | 54605416 |
| 386 | 92957654 | 91.89% | 170829216 | 61.60% | 65562022 |
| 387 | 90627760 | 93.92% | 170231373 | 63.60% | 61998824 |
| 388 | 94159915 | 93.71% | 176478389 | 53.60% | 81955000 |
| 389 | 93895734 | 92.31% | 173357587 | 67% | 57291911 |
| 390 | 93110943 | 91.98% | 171288798 | 65.50% | 59067248 |
| 391 | 91179562 | 93.39% | 170307295 | 60.70% | 66994666 |
| 392 | 94007538 | 94.13% | 176984606 | 57% | 76037137 |
| 393 | 92783466 | 91.90% | 170532129 | 65.30% | 59175784 |
| 394 | 93347423 | 93.30% | 174193583 | 54.10% | 80031876 |
| 395 | 93719900 | 92.07% | 172581611 | 56.80% | 74499816 |
| 396 | 94289838 | 91.92% | 173338731 | 68.30% | 54872025 |
| 397 | 94653775 | 93.44% | 176883243 | 62.80% | 65720019 |
| 398 | 94164370 | 93.32% | 175752605 | 61.90% | 66968050 |
| 399 | 88567436 | 93.30% | 165258210 | 69.10% | 51033967 |
| 400 | 98416526 | 92.21% | 181506660 | 61% | 70857795 |
| 401 | 95476259 | 92.08% | 175822231 | 74% | 45652965 |
| 402 | 92721577 | 93.59% | 173558028 | 66.90% | 57505064 |
| 403 | 92259150 | 91.10% | 168094982 | 66.80% | 55805579 |
| 404 | 88083362 | 93.76% | 165177022 | 58.10% | 69219073 |
| 405 | 92673640 | 92.23% | 170944821 | 68.20% | 54331377 |
| 406 | 91470878 | 90.71% | 165950945 | 68.30% | 52630329 |
| 407 | 92289829 | 91.24% | 168411040 | 66.50% | 56472297 |
| 408 | 88452199 | 92.23% | 163152205 | 63.10% | 60181547 |
| 409 | 94457069 | 91.78% | 173385603 | 69.20% | 53418541 |
| 410 | 90804922 | 90.60% | 164542909 | 59.40% | 66759518 |
| 411 | 92170656 | 92.31% | 170159210 | 58.80% | 70080399 |
| 412 | 88015234 | 90.63% | 159541716 | 67.40% | 52066946 |
| 413 | 90896266 | 92.02% | 167291419 | 66.50% | 55964132 |
| 414 | 95267470 | 93.90% | 178918725 | 63.10% | 66019900 |
| 415 | 90307683 | 89.91% | 162395740 | 75.20% | 40311703 |
| 416 | 89954030 | 93.51% | 168237445 | 72.70% | 45963463 |
| 417 | 92393129 | 90.77% | 167722925 | 58.60% | 69448174 |
| 418 | 96504239 | 92.38% | 178305273 | 54.50% | 81161684 |
| 419 | 94181054 | 92.24% | 173736160 | 53.60% | 80633299 |
| 420 | 88132332 | 92.81% | 163596957 | 55.20% | 73288089 |
| 421 | 92289521 | 93.42% | 172434278 | 56.30% | 75417880 |
| 422 | 93722558 | 91.93% | 172308930 | 59.10% | 70468747 |
| 423 | 91533009 | 90.67% | 165985429 | 72.30% | 45947853 |
| 424 | 98072046 | 92.20% | 180847605 | 66.80% | 59997635 |
| 425 | 94242577 | 88.97% | 167693401 | 61.30% | 64908370 |
| 426 | 90280373 | 91.21% | 164689198 | 66.60% | 54965722 |
| 427 | 93086902 | 94.13% | 175245153 | 62.80% | 65237940 |
| 428 | 88128911 | 92.82% | 163601591 | 63.40% | 59947062 |
| 429 | 93573424 | 93.78% | 175506311 | 62.50% | 65822250 |
| 430 | 93868423 | 93.42% | 175381316 | 57.60% | 74397340 |
| 431 | 98482895 | 90.88% | 179010692 | 73.80% | 46966176 |
| 432 | 93638980 | 93.02% | 174207479 | 64.10% | 62462076 |
| 433 | 89719416 | 93.27% | 167369730 | 65.70% | 57463722 |
| 434 | 95073085 | 91.95% | 174833170 | 61.70% | 66880236 |
| 435 | 89822530 | 92.97% | 167011901 | 67.30% | 54612076 |
| 436 | 91834032 | 92.67% | 170198511 | 61.20% | 66085430 |
| 437 | 92515385 | 89.52% | 165636384 | 71.80% | 46708002 |
| 438 | 81638563 | 93.65% | 152909707 | 55.20% | 68573636 |
| 439 | 100452737 | 93.09% | 187024703 | 54.60% | 84975617 |
| 440 | 96805206 | 92.74% | 179556773 | 65.90% | 61168508 |

| | | | | | |
|---|---|---|---|---|---|
| 441 | 98930632 | 93.51% | 185023729 | 58.70% | 76505368 |
| 442 | 96017904 | 92.77% | 178147884 | 58.20% | 74503587 |
| 443 | 90936815 | 93.73% | 170469363 | 58.90% | 70128609 |
| 444 | 89671949 | 92.11% | 165189706 | 63.80% | 59734038 |
| 445 | 93655808 | 93.56% | 175242283 | 56.20% | 76800194 |
| 446 | 92481706 | 93.39% | 172736906 | 60.80% | 67797725 |
| 447 | 92242548 | 93.29% | 172115150 | 58.20% | 71974570 |
| 448 | 98958108 | 92.46% | 183000445 | 54.90% | 82605208 |
| 449 | 89329760 | 93.54% | 167121757 | 57.40% | 71144290 |
| 450 | 92546113 | 92.53% | 171258949 | 52.70% | 80939003 |
| 451 | 95495914 | 90.44% | 172734328 | 64.70% | 60986570 |
| 452 | 90617866 | 91.86% | 166476607 | 56.70% | 72097969 |
| 453 | 85249239 | 92.77% | 158175937 | 50% | 79019520 |
| 454 | 96473089 | 93.21% | 179838851 | 61.30% | 69667130 |
| 455 | 87622388 | 93.39% | 163658186 | 52.40% | 77843675 |
| 456 | 106753538 | 95.40% | 203694475 | 53.80% | 94184243 |
| 457 | 97876344 | 94.93% | 185836410 | 59.10% | 75983609 |
| 458 | 96724752 | 94.96% | 183708439 | 56.70% | 79591673 |
| 459 | 98803703 | 94.37% | 186480153 | 56.10% | 81804328 |
| 460 | 93016321 | 94.98% | 176700272 | 54% | 81212759 |
| 461 | 92084819 | 94.83% | 174656278 | 55.80% | 77212005 |
| 462 | 95001560 | 93.67% | 177982126 | 57.20% | 76218237 |
| 463 | 106809646 | 94.65% | 202187866 | 57.80% | 85413716 |
| 464 | 87839255 | 94.12% | 165355226 | 52.80% | 77967579 |
| 465 | 105811413 | 94.38% | 199739189 | 56.70% | 86405329 |
| 466 | 95541031 | 91.59% | 175007190 | 69.10% | 54148777 |
| 467 | 100430849 | 94.49% | 189789449 | 58.90% | 77935513 |
| 468 | 105982368 | 93.82% | 198873360 | 56.20% | 87132432 |
| 469 | 101514895 | 94.94% | 192751591 | 50.30% | 95763249 |
| 470 | 94660040 | 89.61% | 169654972 | 59.10% | 69446557 |
| 471 | 101901254 | 95.58% | 194798665 | 54.90% | 87802945 |
| 472 | 94699721 | 94.57% | 179109617 | 57.20% | 76588848 |
| 473 | 105237557 | 94.78% | 199487211 | 54.10% | 91466671 |
| 474 | 95567120 | 96.24% | 183956252 | 62.70% | 68536981 |
| 475 | 94395254 | 96.17% | 181562730 | 58.70% | 74947279 |
| 476 | 98761952 | 94.66% | 186975979 | 58.10% | 78341010 |
| 477 | 92467802 | 94.89% | 175485793 | 59.10% | 71696131 |
| 478 | 96982315 | 94.40% | 183094897 | 61.40% | 70687779 |
| 479 | 101831651 | 94.93% | 193331574 | 53.20% | 90575634 |
| 480 | 97300773 | 95.01% | 184887565 | 52.60% | 87728858 |
| 481 | 96666603 | 93.43% | 180640088 | 63.40% | 66139023 |
| 482 | 102251855 | 95.72% | 195760752 | 58.30% | 81682880 |
| 483 | 97637176 | 94.63% | 184781648 | 52.90% | 86991385 |
| 484 | 101283273 | 93.99% | 190390008 | 57.20% | 81456991 |
| 485 | 101091302 | 94.91% | 191881678 | 57.90% | 80701975 |
| 486 | 93013111 | 94.11% | 175065722 | 59.80% | 70354519 |
| 487 | 105604071 | 94.43% | 199445496 | 54.20% | 91328980 |
| 488 | 93988400 | 93.25% | 175281809 | 64.30% | 62488580 |
| 489 | 99689678 | 94.93% | 189267116 | 59.30% | 77096563 |
| 490 | 103505998 | 95.05% | 196756837 | 58.80% | 80978414 |
| 491 | 97155437 | 94.88% | 184359341 | 61% | 71943927 |
| 492 | 97162311 | 94.64% | 183899317 | 64.20% | 65825221 |
| 493 | 97734107 | 94.45% | 184624265 | 62.60% | 69118740 |
| 494 | 97047216 | 94.80% | 183998443 | 63.20% | 67785009 |
| 495 | 97440239 | 94.48% | 184125148 | 54.40% | 84029016 |
| 496 | 94981444 | 94.62% | 179740485 | 62.30% | 67796232 |
| 497 | 90898018 | 93.98% | 170847926 | 62.10% | 64797984 |
| 498 | 98192837 | 95.43% | 187415265 | 57.50% | 79655358 |
| 499 | 94635900 | 95.09% | 179972994 | 57% | 77352751 |
| 500 | 96304744 | 95.18% | 183331357 | 58.10% | 76877869 |
| 501 | 99152855 | 94.85% | 188094606 | 52.80% | 88830603 |
| 502 | 87912768 | 94.13% | 165498166 | 57.90% | 69747262 |
| 503 | 102072863 | 94.93% | 193786918 | 58.80% | 79862675 |

| | | | | | |
|---|---|---|---|---|---|
| 504 | 95626828 | 94.70% | 181125533 | 62.10% | 68618652 |
| 505 | 95462159 | 95.32% | 181984022 | 58.90% | 74786308 |
| 506 | 86963075 | 94.99% | 165212291 | 59.70% | 66591725 |
| 507 | 102034874 | 94.81% | 193475179 | 62.70% | 72089086 |
| 508 | 93891414 | 94.86% | 178132723 | 61.10% | 69241995 |
| 509 | 95377432 | 94.81% | 180862233 | 61.40% | 69852609 |
| 510 | 105657361 | 94.56% | 199815540 | 59.40% | 81070642 |
| 511 | 94505366 | 95.15% | 179845898 | 54.60% | 81647583 |
| 512 | 93503827 | 95.19% | 178005941 | 92.80% | 12853785 |
| 513 | 97584136 | 94.64% | 184705043 | 55.40% | 82434795 |
| 514 | 94742267 | 94.40% | 178876936 | 57.80% | 75464728 |
| 515 | 96662924 | 95.12% | 183899328 | 60.60% | 72487446 |
| 516 | 95153939 | 94.63% | 180092578 | 60.40% | 71351472 |
| 517 | 94091898 | 93.90% | 176704699 | 62.10% | 66893054 |
| 518 | 92269025 | 94.15% | 173743946 | 59.40% | 70526796 |
| 519 | 94381366 | 94.39% | 178175304 | 56.20% | 77967554 |
| 520 | 95654141 | 95.06% | 181862423 | 58% | 76350924 |
| 521 | 97093342 | 94.96% | 184396026 | 58.60% | 76254633 |
| 522 | 92388585 | 95.13% | 175771350 | 58.80% | 72358164 |
| 523 | 92060426 | 94.69% | 174350333 | 58% | 73313254 |
| 524 | 109881129 | 94.29% | 207219855 | 57.20% | 88731020 |
| 525 | 101050070 | 95.68% | 193365348 | 59% | 79184265 |
| 526 | 96318962 | 94.25% | 181563940 | 58.90% | 74608502 |
| 527 | 91688583 | 94.76% | 173766923 | 58.80% | 71569344 |
| 528 | 98328743 | 95.19% | 187205952 | 58.30% | 78136105 |
| 529 | 121446366 | 94.15% | 228685610 | 57.70% | 96642845 |
| 530 | 100138016 | 94.31% | 188880646 | 61.80% | 72140808 |
| 531 | 94415088 | 95.10% | 179586762 | 57.30% | 76701379 |
| 532 | 102141157 | 95.18% | 194426179 | 55.30% | 86963987 |
| 533 | 97730240 | 95.01% | 185713182 | 54.70% | 84165544 |
| 534 | 96131064 | 95.57% | 183745808 | 55.20% | 82243616 |
| 535 | 95363612 | 94.02% | 179323841 | 59% | 73469130 |
| 536 | 102903390 | 94.13% | 193719440 | 61.20% | 75199767 |
| 537 | 100346986 | 92.46% | 185567738 | 62.10% | 70414077 |
| 538 | 100722120 | 94.73% | 190831813 | 57.70% | 80788804 |
| 539 | 95881211 | 94.30% | 180836733 | 60.70% | 71070593 |
| 540 | 95567678 | 95.28% | 182111290 | 52.60% | 86301224 |
| 541 | 97359400 | 95.05% | 185084691 | 59.30% | 75373328 |
| 542 | 97578199 | 94.91% | 185213389 | 61.30% | 71606892 |
| 543 | 96910187 | 95.58% | 185246359 | 53.70% | 85782395 |
| 544 | 112780954 | 95.18% | 214694984 | 52.20% | 102724183 |
| 545 | 93275663 | 95.35% | 177880631 | 56.20% | 77840263 |
| 546 | 83354267 | 94.64% | 157767241 | 50.80% | 77638649 |
| 547 | 90439586 | 95.09% | 171994479 | 59.60% | 69448972 |
| 548 | 103168200 | 93.69% | 193320598 | 52.20% | 92492657 |
| 549 | 98614058 | 95.08% | 187530774 | 52.90% | 88392266 |
| 550 | 98772349 | 94.43% | 186545174 | 51.80% | 89907393 |
| 551 | 96029438 | 91.99% | 176675593 | 58.20% | 73793839 |
| 552 | 94987817 | 94.13% | 178823254 | 55.70% | 79172902 |
| 553 | 98177356 | 95.20% | 186920472 | 52.80% | 88148676 |
| 554 | 94374784 | 94.06% | 177536940 | 56.20% | 77690572 |
| 555 | 99184295 | 94.78% | 188008498 | 61.80% | 71824464 |
| 556 | 96475347 | 93.23% | 179896780 | 56.40% | 78377785 |
| 557 | 95325137 | 94.82% | 180772579 | 55.80% | 79913858 |
| 558 | 99134429 | 96.11% | 190559341 | 62.80% | 70917976 |
| 559 | 95543580 | 94.65% | 180869530 | 57.70% | 76457595 |
| 560 | 101595656 | 95.06% | 193147151 | 55.40% | 86101711 |
| 561 | 91435512 | 94.70% | 173172445 | 55% | 77845967 |
| 562 | 95844954 | 93.06% | 178382933 | 60% | 71414095 |
| 563 | 93400224 | 94.81% | 177107821 | 55.50% | 78810835 |
| 564 | 97693345 | 93.78% | 183240742 | 55.80% | 81060064 |
| 565 | 111457841 | 94.01% | 209556407 | 54.40% | 95599806 |
| 566 | 100924314 | 94.69% | 191128298 | 60.80% | 74961152 |

| | | | | | |
|---|---|---|---|---|---|
| 567 | 95146106 | 94.99% | 180753766 | 60% | 72328773 |
| 568 | 92308177 | 94.52% | 174506357 | 60.40% | 69065020 |
| 569 | 93055956 | 94.71% | 176261034 | 53.60% | 81708334 |
| 570 | 94736443 | 94.54% | 179120753 | 59.50% | 72515103 |
| 571 | 101645086 | 94.82% | 192751968 | 59.50% | 78029324 |
| 572 | 95245554 | 93.82% | 178711513 | 58.70% | 73752412 |
| 573 | 94462242 | 94.09% | 177753492 | 58.60% | 73591688 |
| 574 | 94920698 | 94.50% | 179406360 | 57.90% | 75451736 |
| 575 | 93789701 | 95.31% | 178776881 | 57.80% | 75530134 |
